# Prefoldin 2 contributes to mitochondrial morphology and function

**DOI:** 10.1186/s12915-023-01695-y

**Published:** 2023-09-12

**Authors:** Ismail Tahmaz, Somayeh Shahmoradi Ghahe, Monika Stasiak, Kamila P. Liput, Katarzyna Jonak, Ulrike Topf

**Affiliations:** Institute of Biochemistry and Biophysics, Polish Academy of Sciences, Pawińskiego 5a, 02-106 Warsaw, Poland

**Keywords:** Prefoldin, Pfd2/Gim4, Tom70, Mitochondria, Chaperone, Proteostasis

## Abstract

**Background:**

Prefoldin is an evolutionarily conserved co-chaperone of the tailless complex polypeptide 1 ring complex (TRiC)/chaperonin containing tailless complex 1 (CCT). The prefoldin complex consists of six subunits that are known to transfer newly produced cytoskeletal proteins to TRiC/CCT for folding polypeptides. Prefoldin function was recently linked to the maintenance of protein homeostasis, suggesting a more general function of the co-chaperone during cellular stress conditions. Prefoldin acts in an adenosine triphosphate (ATP)-independent manner, making it a suitable candidate to operate during stress conditions, such as mitochondrial dysfunction. Mitochondrial function depends on the production of mitochondrial proteins in the cytosol. Mechanisms that sustain cytosolic protein homeostasis are vital for the quality control of proteins destined for the organelle and such mechanisms among others include chaperones.

**Results:**

We analyzed consequences of the loss of prefoldin subunits on the cell proliferation and survival of *Saccharomyces cerevisiae* upon exposure to various cellular stress conditions. We found that prefoldin subunits support cell growth under heat stress. Moreover, prefoldin facilitates the growth of cells under respiratory growth conditions. We showed that mitochondrial morphology and abundance of some respiratory chain complexes was supported by the prefoldin 2 (Pfd2/Gim4) subunit. We also found that Pfd2 interacts with Tom70, a receptor of mitochondrial precursor proteins that are targeted into mitochondria.

**Conclusions:**

Our findings link the cytosolic prefoldin complex to mitochondrial function. Loss of the prefoldin complex subunit Pfd2 results in adaptive cellular responses on the proteome level under physiological conditions suggesting a continuous need of Pfd2 for maintenance of cellular homeostasis. Within this framework, Pfd2 might support mitochondrial function directly as part of the cytosolic quality control system of mitochondrial proteins or indirectly as a component of the protein homeostasis network.

**Supplementary Information:**

The online version contains supplementary material available at 10.1186/s12915-023-01695-y.

## Background

Protein chaperones are essential components within the protein homeostasis network [1, 2]. They are required for folding de novo-produced polypeptides and assist as holdases and disaggregases of misfolded and aggregated proteins during cellular stress conditions. Oligomeric chaperonins are mostly implicated in folding newly synthesized proteins. In eukaryotes, TRiC/CCT together with its co-chaperone prefoldin, assists in folding approximately 10% of polypeptides downstream of the translation machinery [3, 4]. The TRiC/CCT can fold proteins in an adenosine triphosphate (ATP)-dependent manner, whereas the prefoldin complex acts as a holdase, and its function is ATP-independent [5].

Prefoldin consists of six subunits that form a heterohexameric complex that resembles a jellyfish-like shape [6]. It is composed of two α subunits (Pac10/Pfd3 and Gim5/Pfd5) and four β subunits (Pfd1, Gim4/Pfd2, Gim3/Pfd4, and Yke2/Pfd6) [7, 8]. The identification of native substrates of the eukaryotic prefoldin complex is mostly limited to the cytoskeletal proteins actin and tubulin. Thus, our current understanding of the potential role of prefoldin during protein homeostasis maintenance is incomplete [9]. Interestingly, each of the single subunits can bind unique and specific interactors, which might allow them to engage in non-canonical functions [9]. In recent years, mechanistic insights that define non-canonical roles of the prefoldin complex were unraveled [10–12]. The co-chaperone gained attention in the fields of transcription regulation [13–17], protein turnover, and protein quality control [18–20]. Contemporary research indicates that prefoldin could play a role during cellular conditions characterized by a high burden of misfolded or unfolded proteins [18, 21–24].

In the present study, we sought to elucidate the function of prefoldin under cellular stress conditions in the yeast *S. cerevisiae*. In contrast to higher eukaryotes, budding yeast can live without a functional prefoldin complex because cytoskeletal proteins can also fold independently of the co-chaperone [25]. This offers an advantage of using yeast as a model to understand prefoldin function under stress conditions that also limit ATP availability. Changes in the yeast cell environment, such as changes in temperature, oxidative stress, and switches in growth media, cause challenges to cellular protein homeostasis. Moreover, growth in respiratory medium depends on functional mitochondria.

Mitochondrial biogenesis relies largely on nuclear-encoded genes. Mitochondrial proteins must be produced by cytosolic translation machinery, and mitochondrial precursor proteins are imported into the organelle [26, 27]. Precursor proteins need to be kept unfolded before their import into mitochondria. Various cytosolic chaperones have been identified to assist the biogenesis of mitochondrial proteins in the cytosol. For example, chaperones of the Hsp70 and Hsp90 family, as well as some of their co-chaperones, such as Ydj1 and Sti1, respectively, assist the biogenesis of mitochondrial presequence-containing substrates and proteins that are localized to the inner membrane [28–31]. The biogenesis of outer membrane proteins is assisted by the co-chaperones Djp1 and Xdj1 [32, 33], and mitochondrial β-barrel proteins utilize Hsp70, Ydj1, and Sis1 [33, 34]. Thus, under physiological conditions, a network of chaperones and co-chaperones bind mitochondrial precursor proteins, and some were also shown to interact with the outer membrane import complex (TOM) to facilitate the targeting of precursor proteins to mitochondria [32].

Conditions that prevent the import of mitochondrial proteins into the organelle were shown to cause a burden for cellular proteostasis through the accumulation of non-imported mitochondrial proteins [35, 36]. Multiple mechanisms were shown to counteract the proteotoxicity of accumulating mitochondrial proteins, including transcriptional rewiring, alterations of translation, post-translational mechanisms, and segregation of non-imported precursor proteins into storage granules [37–39]. Currently, there is scarce information about the involvement of cytosolic chaperones in managing mitochondrial proteins under cellular stress conditions. We considered the prefoldin complex as a suitable candidate because of its ability to act in an ATP-independent manner and its potential for an extended substrate landscape.

In the present study, we linked the function of the co-chaperone prefoldin to mitochondria by supporting cellular growth under respiratory conditions presumably by contributing to mitochondrial biogenesis. The growth deficiency was increased by concomitant heat stress. We found that the loss of the prefoldin subunit 2 (Pfd2; Gim4 in *S. cerevisiae*) led to a decrease in mitochondrial protein levels connected with some components of the respiratory chain complexes.

## Results

### Prefoldin complex subunits support growth upon exposure to some environmental stressors

**Fig. 1:**
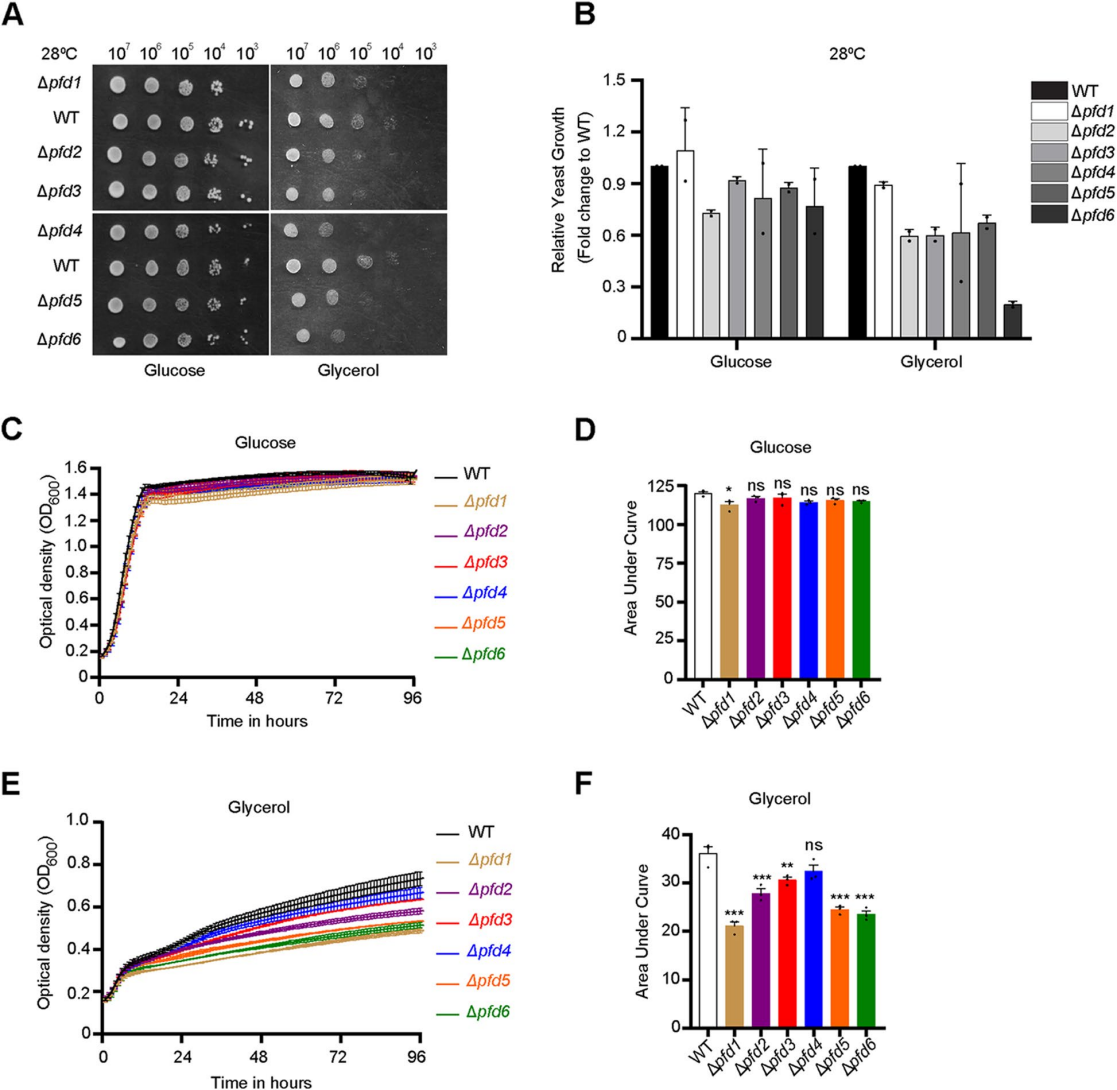
Loss of prefoldin subunits leads to a decrease in growth under respiratory conditions. **A** Ten-fold dilutions of yeast cells of the indicated strains were spotted on solid agar plates with complete synthetic medium that contained glucose or glycerol. Cells were grown at 28 °C for 3 days. Experiments were performed in two biological repetitions. **B** Quantification of spot test shown in **A**. The data are expressed as the mean ± SD. *n* = 2. **C, E** Wildtype cells and yeast cells that lacked single prefoldin subunits were grown on complete synthetic medium that contained glucose (**C**) or glycerol (**E**). The growth of yeast cells was monitored over time. Each experiment was performed in three biological repetitions. The data are expressed as the mean ± SEM. **D, F** Quantification of growth shown in **C** and **E**, respectively, by calculating the area under the curve using GraphPad Prism 8.3.0 software. The data are expressed as the mean ± SEM. *n* = 3. ***p < 0.001, **p < 0.01, *p < 0.05. ns, not significant. WT, wild type

To define cellular stress conditions that entail the function of prefoldin complex subunits, we performed growth tests using various commonly examined stressors. We applied oxidative, osmotic, ultraviolet (UV), and heat stress (Fig. 1, Additional file 1). Single-subunit deletions and wildtype yeast cells were grown on a solid medium. To induce oxidative stress, hydrogen peroxide was added to the plates. Cells that lacked single subunits grew less upon exposure to oxidative stress compared with wildtype cells (Additional file 1: Fig. S1A). In contrast, the growth of cells with single subunit deletions was not more affected than wildtype cells under osmotic stress (Additional file 1: Fig. S1B, C). Interestingly, exposure to UV light, which induces DNA damage, showed that *PFD1* deletion was resistant to this stress compared with wildtype cells while other deletion cells were as affected as wildtype cells upon UV stress (Additional file 1: Fig. S1D).

Exposure to different growth temperatures revealed the sensitivity of single-subunit deletions to extreme temperatures. As shown previously, low temperatures caused a growth defect of cells that lacked prefoldin subunits ([40], Additional file 1: Fig. S1E), but at the moderate temperature of 28 °C, the deletion strains grew similarly to the wildtype strain on fermentative (glucose) medium (Fig. 1A, B). However, a high temperature (37 °C) affected the growth of cells that lacked prefoldin subunits (Additional file 1: Fig. S1E). Especially, the loss of *PFD1*, *PFD4*, *PFD5*, and *PFD6* decreased the cell growth compared with wildtype cells. Forty-two degree Celsius growth temperature nearly abolished the growth of deletion cells compared with wildtype cells when grown on a fermentative (glucose) medium (Additional file 1: Fig. S1E). We concluded that prefoldin function supports the cells’ growth upon stressors that can cause challenges for cellular protein homeostasis, such as an increase in oxidative stress, and heat shock.

We noticed that prefoldin deletions somewhat grew less in respiratory (glycerol) medium compared to wildtype cells at 28 °C (Fig. 1A, B) but growth at high temperature and respiratory medium affected wildtype and deletion cells likewise (Additional file 1: Fig. S1E). We confirmed this slow-growth phenotype in liquid culture. Cells with deletions that were grown in a medium that contained glycerol (Fig. 1E, F) were unable to reach a similar optical density as wildtype cells. Such differences were not observed when growing cells on a glucose-containing medium (Fig. 1C, D). To investigate whether the prefoldin complex is needed to withstand mitochondrial stress, we applied antimycin A, an inhibitor of respiratory chain complex III in mitochondria. Cells that lacked the prefoldin subunits 2, 3, 5, and 6 showed a tendency to grow less compared to wildtype cells upon antimycin A-induced stress (Additional file 2: Fig. S2A, B). Thus, prefoldin appears to be involved in physiological conditions that require mitochondrial function rather than upon mitochondrial stress.

### *PFD2* and *PFD5* support an extended mitochondrial network morphology

**Fig. 2:**
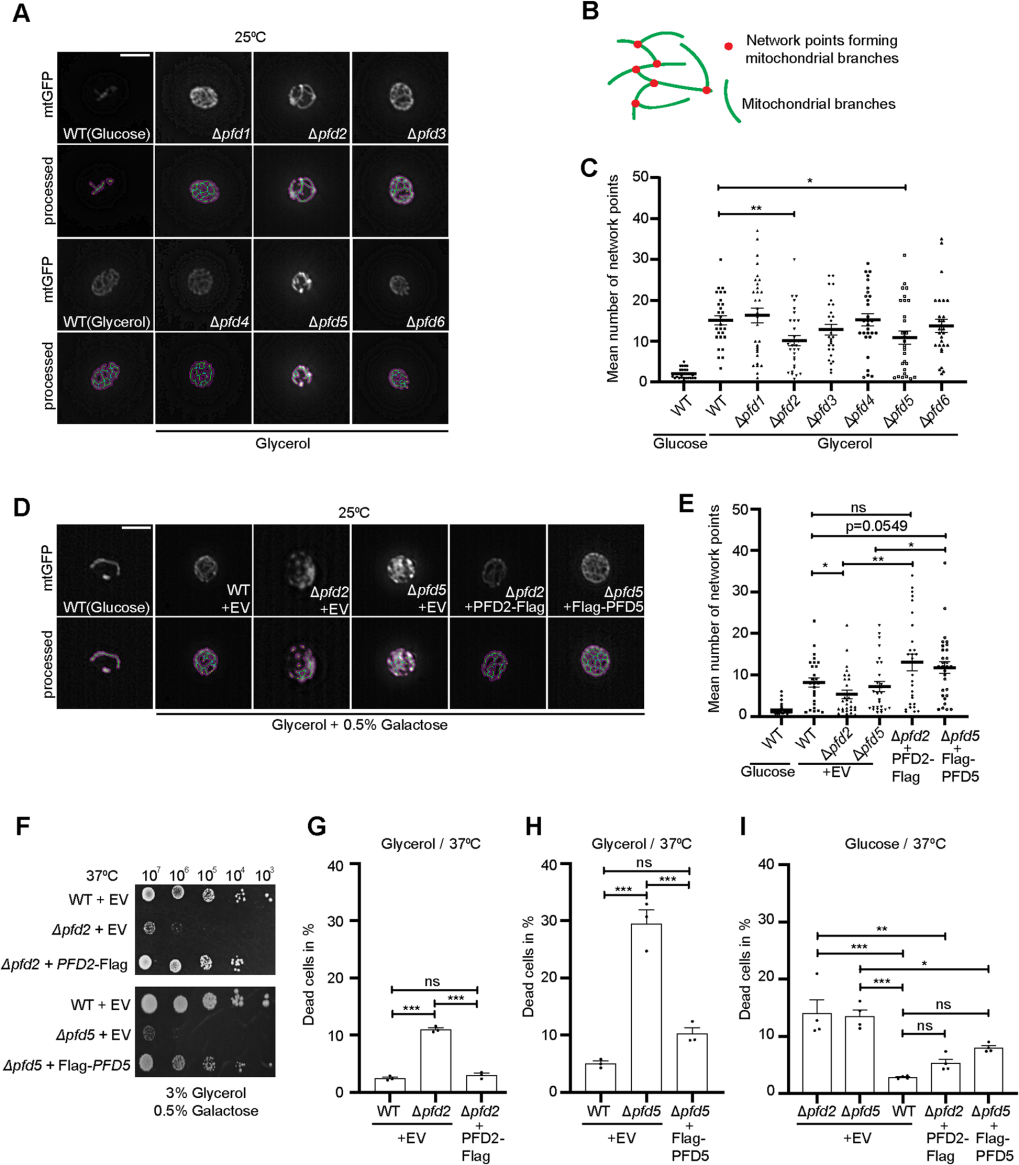
Loss of *PFD2* and *PFD5* results in defective mitochondria network. **A**,** D** Yeast cells that were transformed with a plasmid that harbored mitochondrial-targeted GFP (mtGFP) were grown at 25 °C on selective synthetic medium that contained glycerol or glucose as indicated. Representative images are shown for the expression of mitochondrial GFP and images processed with the MINA plugin tool in ImageJ (see “Methods” section) of the same cell are shown below. Pink lines represent mitochondrial footprint generated by MINA plugin tool. Scale bar = 5 µm. **B** Schematic representation of features taken into account to quantify the number of mitochondrial networks shown in **C** and **E**. **C**, **E** Analysis of mitochondrial morphology, expressed as the number of network points. The data are expressed as the mean ± SEM. *n* = 3. ***p < 0.001, **p < 0.01, *p < 0.05. **D–I** Wildtype cells or cells that lacked *PFD2* or *PFD5* were transformed with an empty vector or a plasmid that harbored *PFD2-Flag* or *Flag-PFD5,* respectively. **F** Ten-fold dilutions of yeast cells of the indicated strains were spotted on selective medium plates that contained glycerol and 0.5% galactose. Cells were grown at 37 °C for 6 days. Experiments were performed in at least two biological repetitions. **G**, **H** Transformed yeast cells were grown in selective **G**, **H** glycerol- or **I** glucose-containing liquid medium to the logarithmic growth phase at 25 °C. Galactose (0.5%) was added to the cultures to induce expression from the plasmid, and cells were shifted to 37 °C for 4 h. Cell viability was assessed by staining with propidium iodide and analyzed by flow cytometry. The data are expressed as the mean ± SEM. *n* = 3. ***p < 0.001; ns, not significant. WT, wild type; EV, empty vector

We chose to further elucidate the relationship between the cytosolic co-chaperone prefoldin and mitochondria. First, we examined mitochondrial morphology. Strains with single chromosomal deletions of prefoldin subunits were transformed with a plasmid that expressed green fluorescent protein (GFP) that was targeted to the mitochondrial matrix. All yeast strains were grown in a respiratory medium at 25 °C to observe phenotypes under physiological conditions. At 25 °C growth temperature, prefoldin subunit deletions 1, 2, 3, 4, and 6 exhibited a higher percentage of dead cells compared to wildtype cells but overall cell viability was still above 90% (Additional file 1: Fig. S1F). The mitochondrial network in cells that lacked *PFD1*, *PFD3*, *PFD4*, and *PFD6* appeared to be similar to wildtype cells. However, we observed a less extended network in cells that lacked *PFD2* and *PFD5* (Fig. 2A). We quantified the overall mitochondrial morphology by assessing the number of network points formed by the branches of the network ([41], Fig. 2B). A higher number of network points indicated a more extended mitochondrial network. The number of network points between wildtype cells and Δ*pfd2* and Δ*pfd5* cells was significantly lower in the deletions indicating disrupted mitochondrial morphology (Fig. 2C). This effect was not caused by an overall lower number of mitochondria within the *PFD2* deletion cells because levels of porin (Por1), a protein of the outer mitochondrial membrane, were equal to protein levels in wildtype cells (Additional file 2: Fig. S2C, D). We noticed that GFP levels varied between the strains and analysis of total protein levels confirmed that cells lacking *PFD2*, *PFD4*, and *PFD5* showed tendencies to higher GFP levels (Additional file 2; Fig. S2E, F). To verify whether GFP is sufficiently imported into the mitochondria total cell lysates (T) of wildtype and Δ*pfd2* cells were fractionated into mitochondria (M) and post-mitochondrial supernatants (S) (Additional file 2: Fig. S2G). Most of the GFP was recovered in the mitochondrial fraction and a minor amount of GFP remained in the post-mitochondrial supernatant proportionally to the total GFP produced (Additional file 2: Fig. S2H). Thus, mislocalized mtGFP did not account for the observed mitochondrial morphology defect in Δ*pfd2* cells.

We investigated whether restoring *PFD2* and *PFD5* function in their respective mutants can rescue the observed mitochondrial morphology phenotype. We expressed *PFD2-*Flag and Flag-*PFD5* under the control of a galactose-inducible promoter. In an independent experiment, we observed and quantified mitochondrial network junction points (Fig. 2D, E). The expression of Flag-tagged versions of *PFD2* and *PFD5* significantly improved mitochondrial morphology (Fig. 2E). Likewise, we investigated if the expression of *PFD2* and *PFD5* improves the growth of the cells with deletions in a respiratory medium when heat shocked. Likely due to the poor growth medium (selective minimal medium), Δ*pfd2* and Δ*pfd5* showed strong growth defect, which was restored upon expression of *PFD2* and *PFD5*, respectively (Fig. 2F). We also investigated whether the decreased growth of the deletion cells under this condition occurs because of a decrease in cell viability. Cells grown in a glycerol-containing liquid medium were shifted to 37 °C for 4 h, and cell viability was assessed by propidium iodide staining and analyzed by flow cytometry. Cells that lacked *PFD2* or *PFD5* showed significantly higher lethality, and supplementation with plasmid-born *PFD2* or *PFD5* decreased the percentage of dead cells to a level comparable with wildtype cells (Fig. 2G, H). Notable, when cells were pre-grown on glucose-containing medium cell lethality of Δ*pfd5* strain was lower (~ 20% less compared to growth on glycerol) but could still be decreased by overexpression of *PFD5*. The percent of dead cells in Δ*pfd2* strain (~ 10%) did not depend on the carbon source (Fig. 2I). Furthermore, we expressed *PFD2* and *PFD5* in respective strains with chromosomal deletions and exposed cells to antimycin A (Additional file 2: Fig. S2I). The complementation of cells with deletions improved their growth under antimycin A-induced mitochondrial stress compared with the cells with deletions expressing empty vector control (EV).

We concluded that the loss of *PFD2* or *PFD5* is linked to defects in mitochondrial morphology under respiratory conditions and that the deficiencies, at least partially, contributed to the diminished growth of the deletion cells at elevated growth temperatures.

### Overall mitochondrial function is maintained in prefoldin deletion strains at physiological temperature

We investigated whether deficiencies in the mitochondrial morphology concomitant affect the function of mitochondria. We assessed mitochondrial membrane potential by staining cells with MitoTracker Red CMXRos, a dye that accumulates in living cells in the mitochondrial matrix depending on the membrane potential (Additional file 3: Fig. S3A). We did not observe significant differences in mitochondrial membrane potential between wildtype and deletion cells. Further, we analyzed total ATP levels in wildtype cells and cells with deletions of *PFD2*, *PFD3*, and *PFD5* (Additional file 3: Fig. S3B, C). Surprisingly, total cellular ATP levels in the *Δpfd2* strain were mildly but significantly elevated compared with wildtype cells when cells were grown on a respiratory medium (Additional file 3: Fig. S3B). This effect was not observed when cells were grown on a fermentative medium (Additional file 3: Fig. S3C) and deletions of *PFD3* and *PFD5* did not show changes in total ATP levels in either growth condition.

Using DAPI staining, we visualized mitochondrial DNA. Mitochondrial DNA was present in all prefoldin deletions (Additional file 3: Fig. 3D). Oxidative phosphorylation and ATP production depend on the correct biogenesis of respiratory chain complexes. Mitochondria encode some subunits of the respiratory chain complex, and the expression of these genes needs to be coordinated with nuclear-encoded genes. Using real-time quantitative polymerase chain reaction (PCR), we amplified the mitochondrial DNA of the *COX1* and *COX3* genes. We found that both genes existed in a higher copy number in cells that lacked *PFD2* compared with wildtype cells (Additional file 3; Fig. S3E, F). In summary, the mitochondrial morphology defect observed in Δ*pfd2* and Δ*pfd5* cells under physiological growth conditions did not negatively affect cellular ATP levels and mitochondrial membrane potential, which is important for the import of mitochondrial proteins produced in the cytosol. Some degree of adaptation on the level of the mitochondrial genome might be possible such as increased mitochondrial DNA copy number to balance potential deficiencies in mitochondrial protein levels that need to be imported into the organelle.

**Fig. 3:**
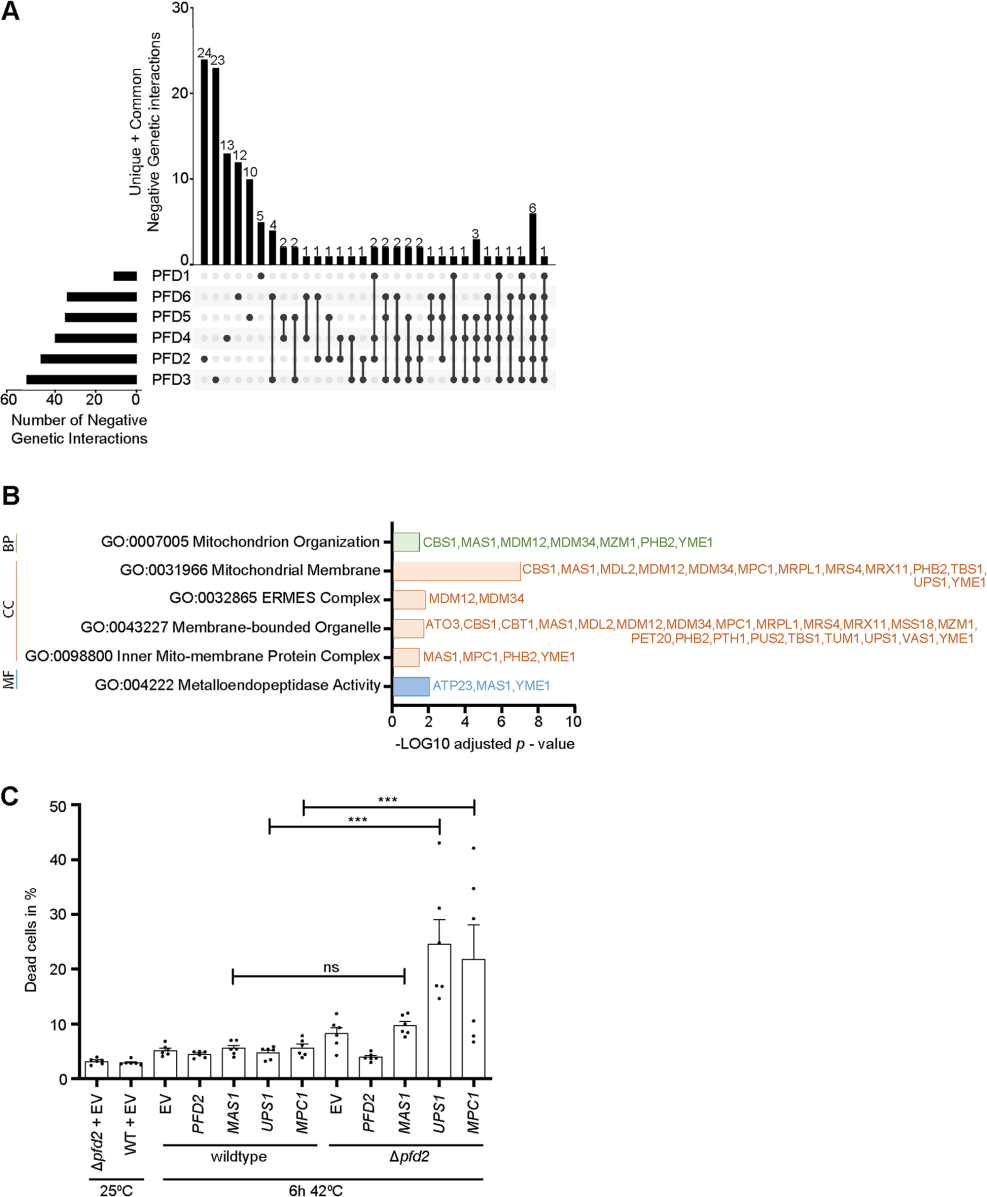
Prefoldin subunits genetically interact with genes that encode mitochondrial proteins. **A** UpSet plot illustrating the number of common and unique mitochondrial genes that showed a negative genetic interaction with single deletions of prefoldin subunits.** B** Gene ontology analysis of mitochondrial genes that showed a negative genetic interaction with *Δpfd2*.** C** Wildtype cells and Δ*pfd2* cells were transformed with an empty vector or a plasmid that harbored mitochondrial genes that were identified as negative genetic interactors with Δ*pfd2.* Gene expression was under control of the constitutive *TEF1* promoter. Transformed strains were grown on selective minimal medium that contained glycerol at 25 °C to the logarithmic growth phase and shifted to 42 °C for 6 h. Cell viability was assessed by propidium iodide staining and analyzed by flow cytometry. The data are expressed as the mean ± SEM. *n* = 4. ***p < 0.001. ns, not significant. WT, wild type

### Loss of *PFD2* causes synthetic sick phenotypes with mitochondrial genes

We hypothesized that prefoldin supports mitochondrial function by influencing the fate of mitochondrial proteins in the cytosol. If mitochondrial proteins are assisted by prefoldin in the cytosol, then the concurrent deletion of prefoldin subunits and mitochondrial genes should result in a synthetic sick phenotype. To address this question, we first analyzed available data from genetic screens (taken from BioGRID interaction repository). A large number of interaction partners were identified with each of the prefoldin subunits. The majority of interactions were genetic interactions. We filtered genetic interaction partners of all prefoldin subunits for only negative genetic interactions (including “synthetic lethality,” “synthetic growth defect,” “phenotypic enhancement,” “synthetic haploinsufficiency,” and “dosage rescue”). A negative genetic interaction refers to a combination of mutations (or deletions) of two genes that results in a more severe fitness phenotype than expected [42]. We found that each of the prefoldin subunits interacts with a unique set of genes: Pfd1 (24), Pfd2 (103), Pfd3 (124), Pfd4 (153), Pfd5 (74), Pfd6 (110) (in brackets are given the numbers of unique interaction partners according to BioGRID interaction repository; Additional file 4: Fig. S4A, Additional file 12: Table S3). When analyzing genetic interaction partners using Gene Ontology (GO) terms, we noticed that distinct GO terms are overrepresented for different prefoldin subunits (Additional file 4: Fig. S4B). For example, *PFD3* deletion results in more severe defects when deleted with genes that encode metal-ion binding proteins. *PFD5* deletion shows a negative interaction with mutations of genes that are related to tRNA metabolism. *PFD6* deletion shows negative interaction when combined with mutations of genes that are related to spliceosome function (Additional file 4: Fig. S4B). Next, we filtered these genes for genes that are associated with mitochondrial function and only analyzed mitochondrial genes that uniquely interact with a certain gene that encodes a single prefoldin subunit (Fig. 3A). Based on these data, we found that all prefoldin subunits form negative genetic interactions with deletions of mitochondrial genes but to different extents (Fig. 3A). *PFD2* deletion showed the largest number of interaction partners. We sought to determine which types of mitochondrial genes these interactions include and performed a GO analysis. The majority of genes were part of the mitochondrial membranes (Fig. 3B). We tested whether the overexpression of mitochondrial genes in cells that lacked *PFD2* restores the growth deficiency of *Δpfd2* cells that are grown on respiratory medium and exposed to high temperature. We focused on genetic interaction partners that clustered in the GO term “Mitochondrial membrane,” which showed the highest statistical significance (Additional file 5: Fig. S5A). We generated constructs that express the mitochondrial genes *MDL2*, *ATP23*, *CBS1*, *MDM34*, *MAS1*, *MTC3*, *MRS4*, *MDM12*, *PHB2*, *MPC1*, and *UPS1* under the constitutively active promoter *TEF1*. Moreover, *PFD2* was also expressed under the *TEF1* promoter and served as a positive control. Plasmids were transformed in cells that lacked *PFD2*, and cell viability was assessed (Additional file 5: Fig. S5B). While the percentage of dead cells in Δ*pfd2* at permissive temperature is low and comparable to wildtype cells, it slightly but reproducibly increased when cells were shifted to higher growth temperature. Plasmid-born *PFD2* expression decreased the percentage of dead cells in *Δpfd2* cells. In contrast, none of the tested mitochondrial proteins improved cell viability. Some of the expressed genes [such as *MPC1* (mitochondrial pyruvate carrier 1) and *UPS1* (phosphatidic acid transfer protein)] even increased the percentage of dead cells to about 20–25% in *Δpfd2* strain (Additional file 5: Fig. S5B). We tested if overexpression of one of the genes [*MAS1* (beta subunit of the mitochondrial processing protease)] results in an increase of the respective protein in the insoluble protein fraction as a potential cause of the decreased cell viability. We found an accumulation of Mas1-HA protein within the insoluble fraction (pellet, P). In contrast, other endogenous proteins that were tested (Rpl17 and Cox4) were detected only in the soluble protein fraction (supernatant, S). However, the formation of Mas1-HA aggregates was found in wildtype cells and *Δpfd2* cells to the same extent (Additional file 5: Fig. S5C, D). To investigate if simply the overexpression of selected mitochondrial genes caused the decrease in cell viability, we performed an independent experiment comparing the percent of dead cells upon overexpression of *MAS1*, *UPS1*, and *MPC1* in wildtype and Δ*pfd2* cells in parallel (Fig. 3C). Ectopic expression of the mitochondrial genes in wildtype cells did not increase the percentage of dead cells compared to empty vector control (EV). In contrast, cell viability was significantly decreased when genes were overexpressed in Δ*pfd2* cells. Thus, the data suggest that cell viability upon overexpression of some mitochondrial genes depends on functional Pfd2 rather than being solely a negative effect of the overproduction of the protein.

### Proteomics analysis reveals the dependents of mitochondrial protein levels on *PFD2* expression

**Fig. 4:**
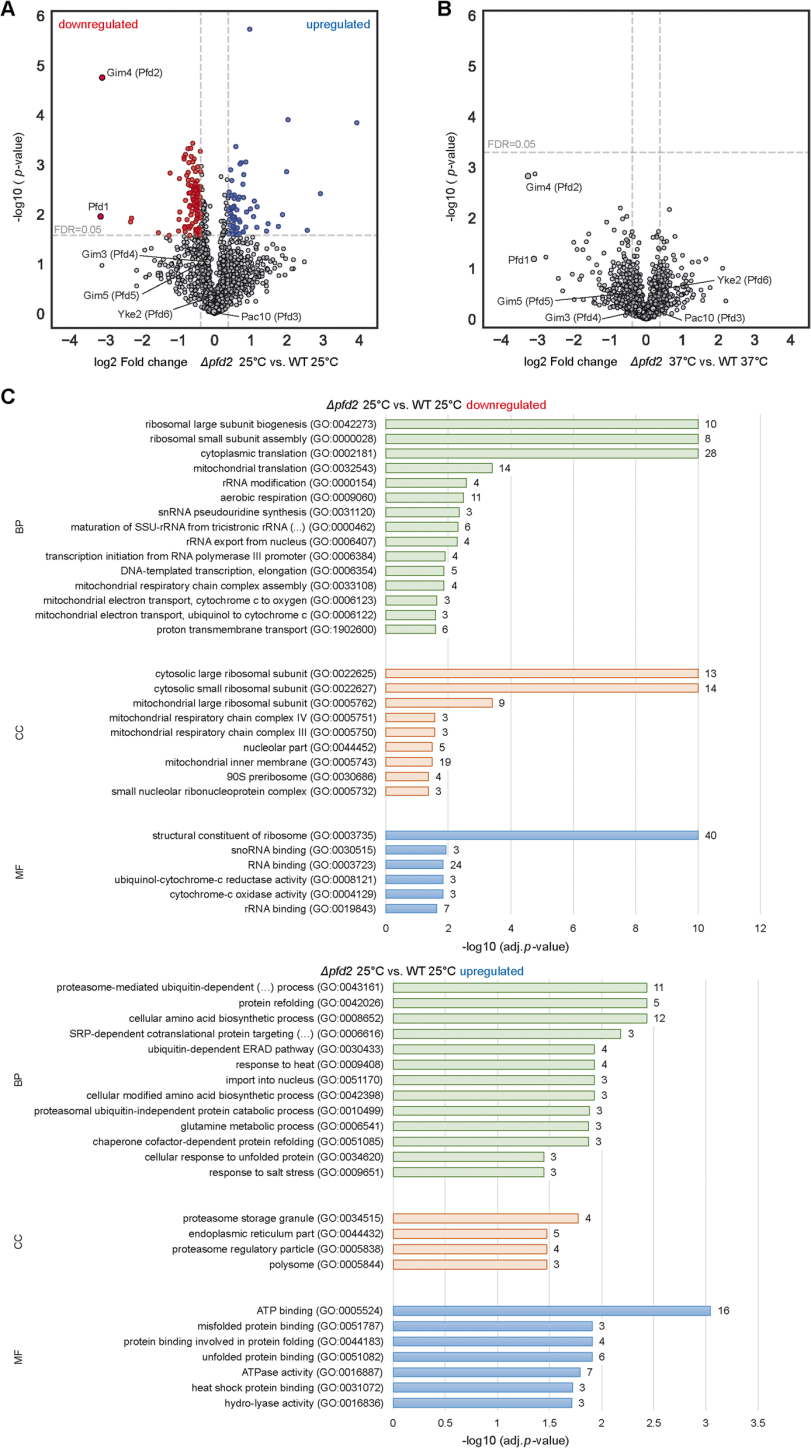
Loss of *PFD2* leads to changes in proteins abundance involved in protein homeostasis. **A, B** Wildtype and *Δpfd2* cells were grown at 25 °C (**A**) and shifted to 37 °C for 4 h (**B**). Volcano plots showing proteins with significantly increased (blue circles) and decreased (red circles) protein abundance in *Δpfd2* versus wildtype cells. Adj. *P*-value of < 0.05 (FDR = 0.05) and fold change of 1.3 was considered as statistical significant change. Non-significant proteins are shown in gray circles. **C** Gene ontology enrichment of proteins with significantly downregulated (upper panel) or upregulated (lower panel) protein abundance in Δ*pfd2* cells compared with wildtype grown at 25 °C. Values next to the right side of the bars indicate the numbers of proteins with the certain GO term. WT, wild type

**Fig. 5:**
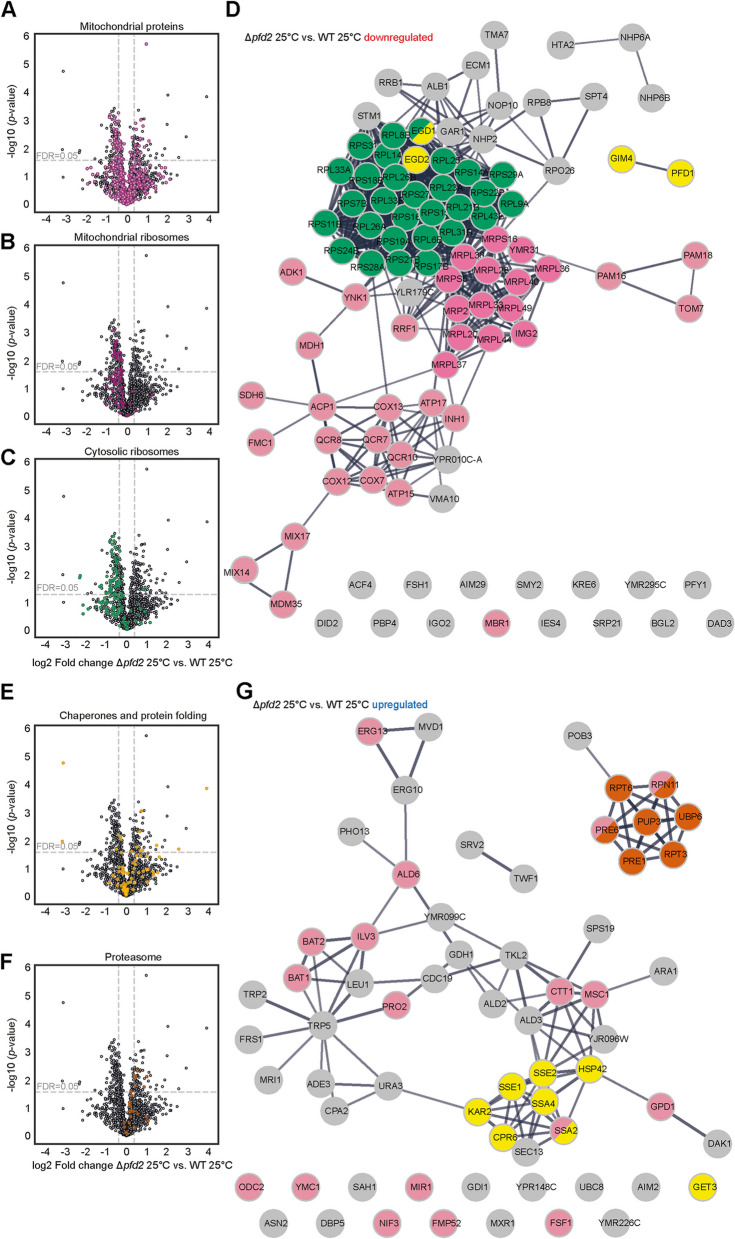
Loss of *PFD2* leads to decreased abundance of mitochondrial proteins at permissive growth temperature. **A–C**,** E**,** F** Volcano plots showing all identified proteins that localize to mitochondria (light pink circles in **A**), mitochondrial ribosome (dark pink circle in **B**), cytosolic ribosome (green circle in **C**), chaperones and protein folding (yellow circle in **E**), and proteasome (brown circle in **F**). **D** Interaction network of all proteins significantly downregulated. In yellow are proteins identified as chaperones and in pink are proteins localized to mitochondria including mitochondrial ribosomes and in green are proteins of the cytosolic ribosome marked.** G** Interaction network of all proteins significantly upregulated. In pink are proteins with mitochondrial localization, in yellow are proteins identified as chaperones and in brown are proteins of the proteasome system marked. WT, wild type

Genetics analysis could only give a partial insight into the importance of Pfd2 for mitochondrial function. Thus, we performed an unbiased proteomics analysis to identify changes in the cellular proteome upon loss of the *PFD2* gene when cells were grown at permissive temperature (25 °C) or exposed to heat shock (4 h 37 °C). Proteins with a fold change of at least 1.3 and an adjusted *p*-value < 0.05 (FDR = 0.05) were considered significantly changed (Additional file 10: Table S1). We first analyzed changes in protein abundance at permissive temperature. We found 100 proteins to be downregulated and 64 proteins upregulated in Δ*pfd2* cells compared with wildtype cells (Fig. 4A). Interestingly, deletion of *PFD2* did not equally affect other subunits of the prefoldin complex (Fig. 4A). Pfd1 subunit was significantly decreased but the protein abundance of the other prefoldin subunits (Pac10/Pfd3, Gim3/Pfd4, Gim5/Pfd5, Yke2/Pfd6) was not significantly changed and thus, the partial assembly of the prefoldin complex cannot be excluded. Gene ontology (GO) enrichment analysis showed that among the significantly downregulated proteins in the *Δpfd2* cells were proteins associated with the cytosolic and mitochondrial translation machinery as well as proteins of the mitochondrial respiratory chain (Fig. 4C and Additional file 11: Table S2). We assigned the cellular localization to all proteins identified and found that proteins with mitochondrial localization showed a tendency to be less abundant in the *Δpfd2* cells compared with wildtype cells (Fig. 5A). However, more striking was the shift of proteins of the mitochondrial ribosome and cytosolic ribosome being generally decreased in the *Δpfd2* cells (Fig. 5B, C). The STRING network of the significantly downregulated proteins showed defined clusters of cytosolic ribosomal proteins, mitochondrial ribosomal proteins, and other mitochondrial proteins most of which were subunits of the respiratory chain (Fig. 5D). The concomitant loss of Pfd1 in the *Δpfd2* cells raised the concern that the decrease in protein abundance could be because of less efficient transcription. Deletion of *PFD1* in yeast was shown previously to affect transcription elongation [13, 43]. To address this concern, we selected proteins of the ribosomes (Rps14A, Rpl21B, Rpl31B, Mrpl20, Mrpl25), respiratory chain complexes (Qcr8, Cox13) and ATPase (Atp5, Atp17), and mitochondrial import (Tom7, Pam16, Pam17) that were downregulated in Δ*pfd2* cells. We analyzed their mRNA levels by quantitative real-time PCR in wildtype, Δ*pfd1* and Δ*pfd2* cells (Additional file 6: Fig. S6A). We did not observe a decrease in transcript levels for any of the genes in Δ*pfd1* and Δ*pfd2* cells compared with wildtype cells but rather a tendency for increased transcript levels in Δ*pfd1* cells indicating that most likely a response on the transcript levels can be excluded as a cause of the decreased protein levels in Δ*pfd2* cells.

Among the significantly upregulated proteins in *Δpfd2* cells were proteins associated with the ubiquitin–proteasome system and proteins involved in protein folding (Fig. 4C and Additional file 11: Table S2). While we did not observe an overall strong tendency for chaperones or the proteasome system to be upregulated (Fig. 5E, F), associated proteins formed distinct clusters in the network analysis (Fig. 5G).

Surprisingly, shifting cells to higher temperatures did not reveal any significant change in protein abundance comparing Δ*pfd2* with wildtype cells (Fig. 4B, Additional file 6: Fig. 6B–F and Additional file 10: Table S1). Proteins of the mitochondrial ribosome remained tending downregulated (Additional file 6: Fig. S6C) and proteins of the proteasome tended to be upregulated (Additional file 6: Fig. S6F). To explain the lack of significant changes between the strains, we analyzed the effect of heat shock individually on wildtype cells and Δ*pfd2* cells (Additional file 7). We found 39 proteins significantly downregulated and 53 proteins significantly upregulated in wildtype cells (Additional file 7: Fig. S7A), whereas in Δ*pfd2* cells 14 proteins were down- and 31 proteins were significantly upregulated (Additional file 7: Fig. S7B). To determine the function of proteins whose production changed in response to the temperature shift, we performed GO term enrichment analysis (Additional file 8, Additional file 9). In wildtype cells, we observed the downregulation of proteins associated with cytoplasmic translation and upregulation of metabolic processes, proteins localizing to mitochondria, and proteins associated with folding (Additional file 8: Fig. S8A). In Δ*pfd2* cells, membrane proteins with transport activity were downregulated and proteins with chaperone activity were upregulated (Additional file 9: Fig. S9A). Generally, in wildtype cells proteins associated with mitochondrial and cytosolic ribosomes tended to be decreased upon higher temperature (Additional file 7: Fig. S7D, E, Additional file 8: Fig. S8B) similarly as in Δ*pfd2* cells at permissive temperature (Fig. 5B, C). This was not observed in the cells with deletions when shifted to a higher temperature (Additional file 7: Fig. S7I, J, Additional file 9: Fig. S9B). Other protein classes of interest (proteins with mitochondrial localization, chaperones and protein folding, proteasome) did not show a clear tendency to be regulated in our experimental condition (Additional file 7: Fig. S7C, F, G and Fig. S7H, K, L). Notable was the regulation of some of the chaperones. In Δ*pfd2* already at permissive temperature, several chaperones including members of the Hsp90 family (Sse1, Cpr6), Hsp70 family (Ssa2, Ssa4), Hsp110 (Sse2), and small heat shock proteins (Hsp42) were upregulated (Fig. 5G). The increase in temperature resulted in upregulation of a similar set of chaperones in wildtype cells [small heat shock proteins (Hsp42, Hsp26), Hsp70 family (Ssa3), Hsp40 family (Mdj1), regulator of Hsp90 (Hch1), and mitochondrial chaperone Hsp60] (Additional file 8: Fig. S8C). Interestingly, in Δ*pfd2* cells upon heat shock, cytosolic co-chaperones Ydj1 and Sti1, and the mitochondrial co-chaperone Hsp10 and chaperone Hsp78 were significantly increased (Additional file 9: Fig. S9C). All four proteins support mitochondrial function [28, 30].

**Fig. 6:**
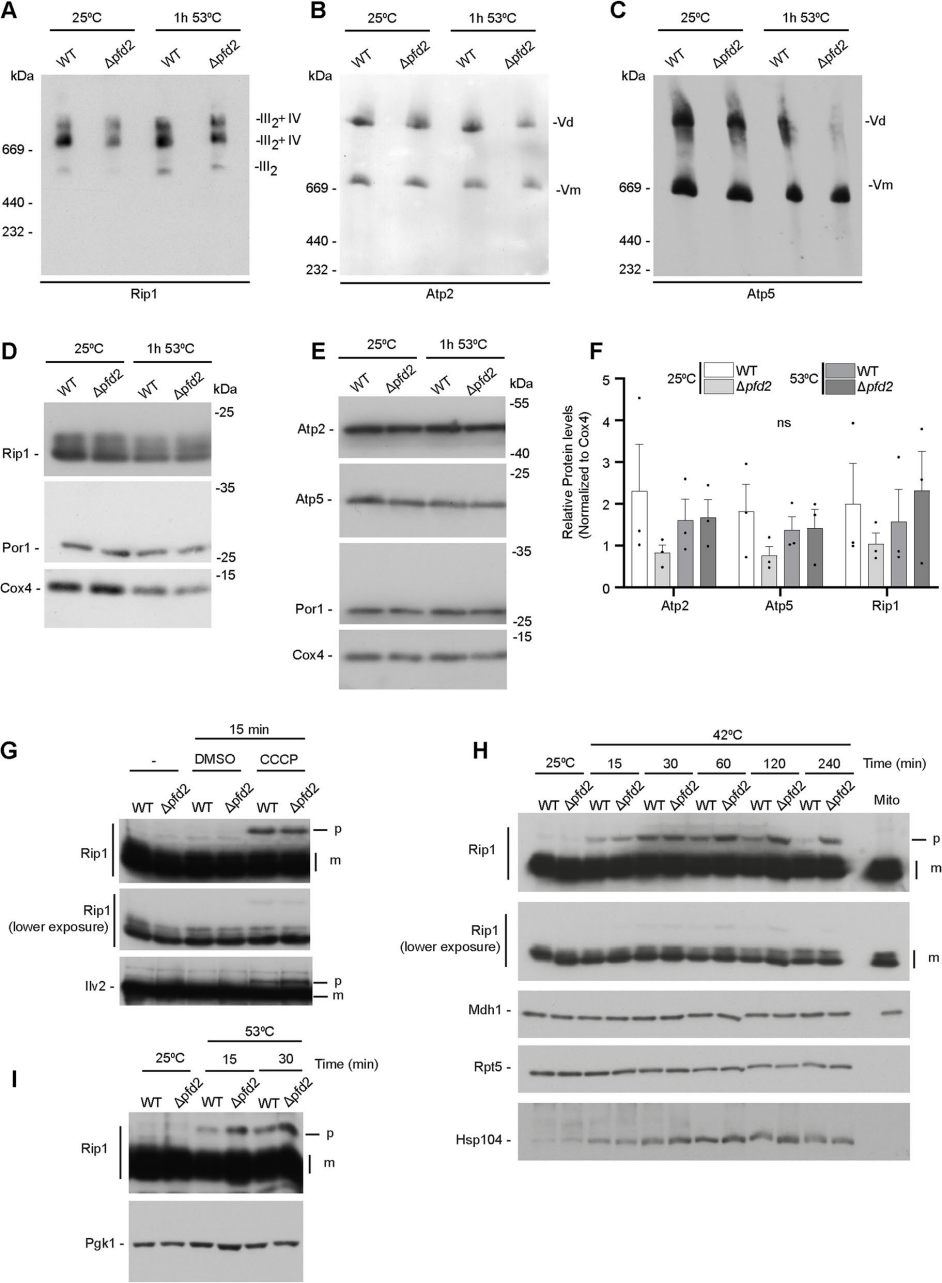
Loss of *PFD2* results in mild deficiencies in respiratory chain complexes. **A–E** Yeast cells were grown in complete synthetic medium that contained glycerol at 25 °C and shifted to 53 °C for 1 h where indicated. Mitochondria were isolated for both growth conditions. **A–C** Mitochondrial extracts were separated on native gel and analyzed by Western blot using specific antibodies. Experiments were performed in three biological repetitions. **D, E** Mitochondrial extracts were separated on SDS-PAGE and analyzed by Western blot using specific antibodies. Blots shown in **D** serve as loading control for native gel shown in **A**. Blots shown in **E** serve as loading control for native gels shown in **B** and **C**. **F** Quantification of mitochondrial protein levels. The data are expressed as the mean ± SEM. *n* = 3. **G–I** Yeast cells were grown in galactose-containing medium at 25 °C and shifted to higher temperature as indicated. Total protein extracts were separated on SDS-PAGE and analyzed by Western blot using specific antibodies. **G** Yeast cells were treated with 40 µM CCCP or an equivalent volume of DMSO (solvent) for 15 min. Cells were harvested before treatment (-) or after treatment (DMSO or CCCP). WT, wild type. Uncropped blots are presented as source data in the Additional file 13

We concluded that loss of *PFD2* stimulated cellular responses of the protein homeostasis network already at permissive growth temperature, while similar responses became activated only upon heat stress in wildtype cells. Therefore, our data provide evidence that Pfd2 function supports cellular protein homeostasis continuously influencing indirectly or directly mitochondrial function.

### Loss of *PFD2* leads to mild deficiencies in the respiratory chain complexes

Since the proteomics data revealed a decrease in protein levels of the respiratory chain in Δ*pfd2* cells at permissive temperature (Fig. 5D), we sought to investigate the abundance and assembly of respiratory chain complexes and ATP synthase (Fig. 6A–C). Mitochondria from cells grown at 25 °C were isolated and digitonin-treated extracts were analyzed by Blue Native electrophoresis. Cells that lacked *PFD2* exhibited a mild decrease in complex III abundance and of super-complexes that were composed of complexes III and IV (Fig. 6A). The ATPase complex was little affected showing only a small decrease in the ATPase dimer levels (Fig. 6B, C). It was previously shown that exposing cells to a high temperature leads to an increase in the assembly of respiratory chain complexes to maintain energy production [44, 45]. This phenomenon was also reflected in our wildtype cells which showed a tendency for increased abundance of complex III and IV when they were exposed to 1 h of heat shock at 53 °C (Fig. 6A). Cells that lacked *PFD2* showed also increased assembly but to a lesser extent compared with wildtype cells (Fig. 6A). The abundance of the ATPase dimer was slightly decreased upon shift to high temperature when probed with an antibody against Atp2, which recognized a beta subunit of the F1 sector (Fig. 6B). Surprisingly, this effect was stronger when probed with an antibody against Atp5 (OSCP; Oligomycin sensitive conferral protein) (Fig. 6C). Atp5 protein is part of the stalk of the ATPase. To control equal loading on the native electrophoresis gels, we analyzed mitochondrial protein content on denaturing gels (Fig. 6D, E). We noted a tendency of decreased levels of Atp2, Atp5, and Rip1 in Δ*pfd2* cells at permissive temperature (25 °C) (Fig. 6F) but generally, levels of these proteins remained similar to wildtype cells and, thus, likely did not account for the observed changes in complex abundance. However, considering the observed decrease in other subunits of the respiratory chain in the proteomics data (see Fig. 5D), we cannot exclude that the abundance of other subunits or proteins that assist the complex assembly contributed to the observed deficits. The deficiencies in the abundance of respiratory chain subunits and ATPase complex were accompanied by a decreasing trend in cell survival in the cells with prefoldin deletions compared with wildtype cells (Additional file 1: Fig. S1G).

Respiratory chain biogenesis depends on proteins produced on cytosolic ribosomes. The precursor proteins need to be transported into the mitochondria. The accumulation of mitochondrial precursor proteins in the cytosol could have several causes including dissipation of membrane potential, diminished import capacity, or decreased degradation of mislocalized precursor proteins. Rip1 precursor protein (pRip1) was below the detection limit in cells grown at permissive temperature. Treating cells with the mitochondrial uncoupler CCCP induced the accumulation of Rip1 precursor in wildtype and Δ*pfd2* cells to a similar extent (Fig. 6G). Interestingly, shifting cells to higher growth temperatures led to detectable accumulation of the Rip1 precursor form (Fig. 6H, I). In a time-dependent manner, pRip1 decreased in wildtype cells but remained at higher abundance in Δ*pfd2* cells when cells were shifted to 42 °C (Fig. 6H). Similarly, shifting cells to 53 °C led to increased accumulation of pRip1 in Δ*pfd2* compared to wildtype cells (Fig. 6I). While the experiments were limited in determining the primary cause of the accumulation of pRip1 (e.g., decreased mitochondrial import or reduced cytosolic removal of pRip1), Pfd2 function might be involved in directing the fate of precursor proteins in the cytosol under cellular stress conditions.

### Pfd2 interacts with a component of the TOM channel

**Fig. 7:**
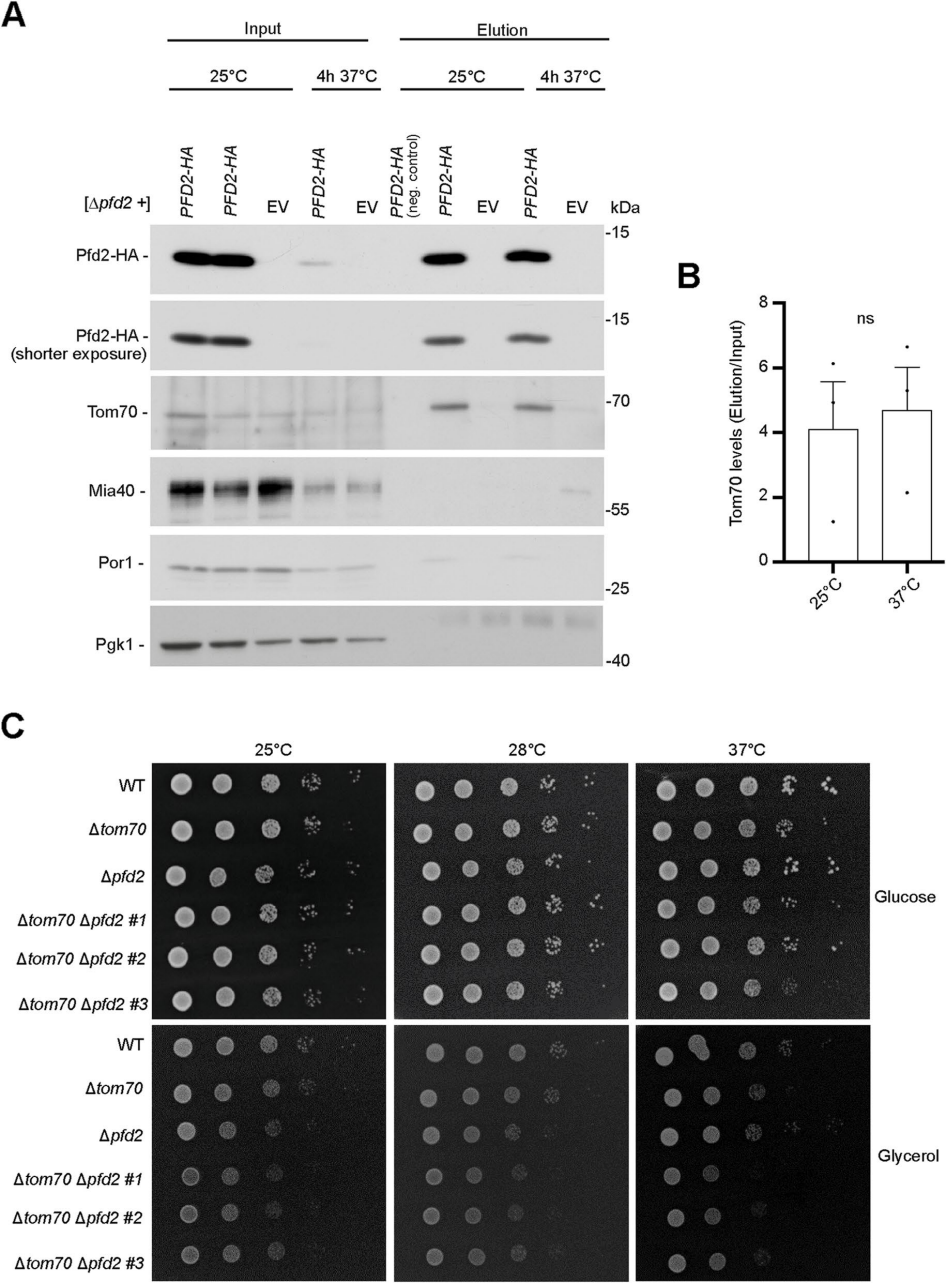
Pfd2 physically interacts with Tom70. **A** Cells that lacked *PFD2* were transformed with a plasmid that expressed *PFD2-HA* or an empty vector. Cells were grown on selective minimal medium that contained glycerol at 25 °C to the logarithmic growth phase and shifted to 37 °C for 4 h. Equal volume of the input and elution fractions of the immunoprecipitation experiment was separated on SDS-PAGE and analyzed by Western blot against specific antibodies. The experiment was repeated three times. “neg. control” (negative control) in the elution fraction indicates that the lysate was incubated with beads not coupled with antibodies. Uncropped blots are presented as source data in the Additional file 13. **B** Quantification of signal for Tom70 in the elution fraction. The data are expressed as the mean ± SEM. *n* = 3. **C** Ten-fold dilutions of wildtype cells, *Δtom70, Δpfd2* and double deletion of *Δtom70 Δpfd2* were spotted on minimal medium plates containing glycerol or glucose. Cells were grown at indicated temperatures for 2–5 days. For the double deletion three different strains (#1, #2, #3) are shown that were maintained after genetic manipulation. Experiment was performed twice with each two technical repetitions with consistent results. WT, wild type

Cytosolic chaperones are needed to guide proteins to the mitochondrial surface where they bind to the translocase of the outer membrane (TOM) by interacting with Tom70, a receptor for incoming precursor proteins [32]. We sought to test if Pfd2 could have played a role during this process. We expressed *PFD2*-HA (hemagglutinin) in *Δpfd2* cells and performed a co-immunoprecipitation experiment (Fig. 7A). We found that Tom70 co-immunoprecipitates with Pfd2-HA. The interaction was present at a growth temperature of 25 °C and remained when the cells were shifted to 37 °C for 4 h (Fig. 7B). We did not find enrichment of the outer membrane protein Por1 (porin) in the elution fraction indicating that Pfd2-HA did not non-specifically interact with the outer membrane. Neither had we found an interaction with Mia40, a protein of the intermembrane space. We created a double deletion of *PFD2* and *TOM70* to analyze a possible genetic interaction between the two deletions (Fig. 7C). Three strains were maintained after genetic manipulations (#1, #2, #3). Strains grew comparable on fermentative medium (glucose). Under respiratory conditions (glycerol), single deletions grew slightly less compared to wildtype cells at 28 °C as expected. At a growth temperature of 37 °C, the double deletions grew visibly less compared to single deletions and wildtype cells suggesting that concomitant loss of *PFD2* and *TOM70* led to a synthetic growth defect. In conclusion, our findings suggest that Pfd2 can interact either directly or indirectly with the receptor of the TOM channel, the entry gate for mitochondrial precursor proteins.

## Discussion

In the present study, we showed that the cytosolic co-chaperone prefoldin supports yeast growth under physiological conditions that require mitochondrial function, upon heat stress and mitochondrial-derived stress but to a smaller extent. Our findings support a continuous involvement of the complex subunit Pfd2 in these processes. However, a mechanism determining if Pfd2 can influence mitochondrial biogenesis directly remains to be shown.

Protein homeostasis is a prerequisite for functional cells and a healthy organism. Failure to maintain protein homeostasis can result in diseases and is a hallmark of aging [46–48]. Dysregulation of prefoldin subunits occurs in various types of cancer cells, which rapidly proliferate and have a high demand for proteome maintenance and high cellular energy consumption, underlining the importance of prefoldin function in pathology [11]. The protein homeostasis network is complex and interconnected. In addition to protein production and degradation, protein folding and the prevention of aggregate formation are essential pillars of protein homeostasis. Protein chaperones and co-chaperones are the workforces that engage in the folding of newly synthesized proteins, unfolding of misfolded proteins, and guidance of misfolded/aggregated proteins toward degradation [49]. The heterohexameric prefoldin complex is an ancient co-chaperone that is known in eukaryotes to hand polypeptides of cytoskeletal proteins over to the chaperonin TRiC/CCT. This canonical function of prefoldin was complemented by accumulating findings that involve the prefoldin complex and its single subunits in transcriptional processes in the nucleus [43]. Moreover, prefoldin was found to co-localize with protein aggregates [18, 19, 50]. Thus, it is unsurprising that the prefoldin complex is involved in processes withstanding heat and oxidative stress, two conditions that can cause protein aggregation. Our findings are consistent with the notion that heat stress limits the growth of yeast cells that lack the Pfd5 subunit [22]. Here, we found that all single prefoldin deletions were sensitive to heat, suggesting that the entire prefoldin complex is necessary to withstand this stress condition. This is in contrast to oxidative stress. Predominantly, Pfd1, Pfd3, and Pfd4 were previously shown to have a transcriptional function that leads to an increase in the expression of oxidative stress defense genes [23]. Interestingly, we found that all prefoldin subunit deletions were limited in growth under respiratory conditions although to different extents. Such growth conditions require an increase in mitochondrial function. We focused our analysis on the Pfd2 subunit, which when deleted showed consistent impairments in mitochondrial morphology. However, our data cannot exclude that other prefoldin subunits can be equally important for the observed mitochondrial phenotypes or complement the Pfd2 function. Occasionally, prefoldin subunits were shown to act independently of its complex [19, 51]. Moreover, biochemical analysis showed that Pfd2 with Pfd3 and Pfd5 with Pfd6 can form stable subcomplexes [9]. Prefoldin-2 (PFDN2) was found to be necessary for modulation of the expression of α-synuclein (SNCG) in retinal ganglion cells, and GO analysis indicated shared mitochondrial function that is associated with *Sncg* and *Pfdn2* in mouse retinal ganglion cells [52]. Furthermore, the disruption of the interaction of the prefoldin complex with its chaperonin TRiC/CCT caused the aggregation of proteins including mitochondrial proteins [22]. Further research will need to show to what extent the TRiC/CCT complex is involved in mitochondrial function under physiological conditions or this function only depends on the prefoldin complex and its subunits.

Our proteomics data suggested that lack of Pfd2 or instability of the prefoldin complex triggers adaptation of the cytosolic and mitochondrial translation machinery. Protein production in the cytosol and mitochondrion was shown to be co-regulated [53]. Thus, the observed decrease in mitochondrial proteins encoded in the nucleus could be a result of decreased translation and this needs to be addressed in further experiments. Further, we cannot exclude that an unstable or not fully assembled prefoldin complex triggers mitochondrial dysfunction indirectly. Several mechanisms were identified to be activated upon mitochondrial dysfunction. This includes the storage of mislocalized mitochondrial proteins in granules to suppress cytotoxicity [39]. In addition, mitochondrial dysfunction triggers the activation of the transcription factor Rpn4, which is necessary for the upregulation of subunits of the proteasome [54]. On the other hand, it was intriguing that the abundance of heat shock proteins normally activated upon increase in temperature were elevated in Δ*pfd2* cells grown at permissive temperature suggesting that cells are already under stress.

Moreover, our data suggest a possible scenario where Pfd2 may act as a holdase of mitochondrial precursor proteins that are produced on cytosolic ribosomes and need to be imported into the organelle. Charged residues at the tips of the coiled coil structure of prefoldin subunits are responsible for substrate binding, and defined residues in the substrate are necessary for the interaction [9]. Specific mitochondrial substrates of Pfd2 remain to be discovered but we demonstrated that Pfd2 can interact with Tom70, the receptor of mitochondrial precursor proteins on the outer mitochondrial membrane. Interestingly, impairments in mitochondrial import were previously shown to significantly increase Pfd2 transcript and protein levels [54]. Other prefoldin subunits were detected, but they were not significantly changed. Notably, Pfd2-HA levels decreased upon heat stress but the interaction between Pfd2 and Tom70 remained under the stress conditions suggesting a continuous need for it. A recent proteomics study showed that prefoldin subunits together with translation machinery were downregulated upon heat stress [55]. Further research will need to show if the interaction with Tom70 is mediated by other chaperones such as Hsp90 or Hsp70 family members.

## Conclusions

In summary, our data showed that Pfd2, a subunit of the cytosolic co-chaperone prefoldin, supports normal mitochondrial morphology and respiratory growth. Our data support a more direct involvement of the prefoldin complex in mitochondrial biogenesis possibly through a mechanism that involves an interaction with Tom70, a receptor of mitochondrial precursor proteins on the outer mitochondrial membrane. Our findings strengthen the role of the prefoldin complex within the proteostasis network and open new directions to explore its function under physiological conditions and in pathology.

## Methods

### Yeast strains and growth conditions

In the present study, wildtype BY4741 and derivative Δ*tom70*, *Δpfd1*, *Δpfd2*, *Δpfd3*, *Δpfd4*, *Δpfd5*, and *Δpfd6* strains were used. All strains used and generated in this study are listed in Table 1. Strains with chromosomal deletions were verified by amplification of the KanMX cassette. Yeast strain carrying the double deletion of *TOM70* and *PFD2* was generated by the replacement of *PFD2* gene with Ura3 cassette amplified from pRS306 vector [56, 57] in a BY4741 strain carrying a single *TOM70* deletion. Deletion of *TOM70* and *PFD2* was checked by PCR, sequencing and qPCR (Δ*pfd2*), or immunoblotting (Δ*tom70*). Three transformants (#1, #2, #3) were saved and used for further experimentation. Yeast strains were maintained on solid YPD (1% [w/v] yeast extract, 2% [w/v] bactopeptone, 2% [w/v] glucose) plates. Yeast cells were generally grown to an optical density at 600 nm (OD_600_) of around 0.6–1.0 at the indicated temperatures in YPG (1% [w/v] yeast extract, 2% [w/v] bactopeptone, 3% [v/v] glycerol, pH 5.2) medium or complete synthetic medium (CSM; 0.67% [w/v] yeast nitrogen base, 0.079% [w/v] CSM amino acid mix) that contained a respiratory carbon source (3% [v/v] glycerol). For analysis of precursor proteins by Western blot, yeast cells were grown on full medium containing 2% [v/v] galactose to an OD_600_ of around 1–2 at 25 °C and shifted to higher temperatures as indicated in the respective figures. Growth tests on solid plates were performed using CSM that contained a fermentative (2% [w/v] glucose) or respiratory (3% [v/v] glycerol) carbon source at 23, 28, 37, and 42 °C. Stress assays were performed by the addition of hydrogen peroxide (catalog no. H1009, Sigma), sodium chloride (catalog no. 117941206, Chempur), mannitol (catalog no. MAN509.5, Bioshop), or sorbitol (catalog no. SOR508.5, Bioshop) to CSM plates that contained 2% glucose (w/v). To check antimycin A-induced stress, yeast strains were plated on YPGal (1% [w/v] yeast extract, 2% [w/v] bactopeptone, 2% [w/v] galactose) plates that contained antimycin A (catalog no. A8674, Sigma). Control plates contained an equivalent amount of ethanol, which was used as the solvent. Ultraviolet irradiation was performed by exposing yeast that were seeded on YPD plates to UV light for 75 s using a UV crosslinker device (CL1000, UVP) as units of J/m^2^. All plates were incubated at 28 °C for 2–3 days. Growth tests in liquid culture were performed in a 96-well plate. Therefore, strains were first grown to stationary in liquid YPD medium and were diluted to an OD600 of ~ 0.2 OD600 in in synthetic minimal media containing either 2% glucose or 3% glycerol yeast cells were grown at 28 °C and growth were monitored in Synergy H1 microplate reader. Optical density at 600 nm for each strain was measured every 60 min for 96 h.

**Table 1:** *Saccharomyces cerevisiae* BY4741 strains used in this study

Strain name	Genotype^a^	Source
***Δpfd1***	*Δpfd1::KanMX4*	Euroscarf
***Δpfd2***	*Δpfd2::KanMX4*	Euroscarf
***Δpfd3***	*Δpfd3::KanMX4*	Euroscarf
***Δpfd4***	*Δpfd4::KanMX4*	Euroscarf
***Δpfd5***	*Δpfd5::KanMX4*	Euroscarf
***Δpfd6***	*Δpfd6::KanMX4*	Euroscarf
***Δtom70***	*Δtom70::KanMX4*	Horizon Discovery/ Yeast knockout collection
***Δtom70 Δpfd2***	*Δtom70::KanMX4 Δpfd2::URA3*	This study

^a^All BY4741 strains are haploid with the background *MATa his3Δ1 leu2Δ0 met15Δ0 ura3Δ0*

To induce the expression of *PFD2*-FLAG or FLAG-*PFD5* from plasmid in cells that were seeded on solid plates (CSM-URA drop-out media that contained 3% [v/v] glycerol), galactose was added to the plates at a final concentration of 0.5% (w/v) and grown at 37 °C for 6–7 days. Expression in liquid medium (CSM that contained 3% [v/v] glycerol and 0.05% [w/v] sucrose) was performed for 4 h after switching the temperature from 25 to 37 °C. Yeast strains that were transformed with a plasmid that harbored mitochondrial-targeted GFP (pYX122) were grown in CSM–HIS drop-out media with 3% (v/v) glycerol and 0.05% (w/v) glucose at 25 °C to monitor mitochondrial morphology.

### Construct generation

To generate pIT03 and pIT07 plasmids, open reading frames of *PFD2* and *PFD5* were amplified from genomic DNA. The receiving plasmid, pESC-URA, was linearized using EcoRI and NotI restriction sites. Cloning resulted in Flag-tagged versions of genes where PFD2 was tagged on the C-terminus, and PFD5 was tagged on the N-terminus.

Genes that gave rise to mitochondrial-targeted proteins were amplified from genomic DNA (see also Table 1), and 1xHA-tag was added at the C-terminus. The receiving plasmid (episomal plasmid [pRS426] that contained the constitutive promoter *TEF1*) was linearized using EcoRI and HindIII restriction enzymes. For all constructs, the PCR products were added to the linearized plasmid by enzyme-free cloning [58]. A complete list of resulting constructs and primers that were used is presented in Table 2.

**Table 2:** Constructs generated in this study

Gene name	Forward primer 5′3′	Reverse primer 5′3′	Plasmid identifier
Cloned in pESC-URA			
***PFD2*** **-Flag**	CAAAAAAAAAGTAAGAATTTTTGAAAATTCCAGCTATGGAACAAAGGAACAACG	CGTCATCCTTGTAATCCATCGATACTAGTGCGTTTTTAACGACTTGAATC	pIT03
**Flag-** ***PFD5***	GAATTTTTGAAAATTCGTAAAAATCAAGCCATGGATTACAAGGATGACGACGATAAGTCCTCTCAAAAAAGTATG	CATCGATACTAGTGCCTAGGCTGTAGACGACTC	pIT07
Cloned in pRS426			
***ATP23***	GGATCCCCCGGGCTGCAGGCAATTATCATTTATGACGATGC	CGAGGTCGACGGTATCGATATCAAGCGTAATCTGGAACATCGTATGGGTATCTGTAAATCTCATCAAACG	pMS018
***MDL2***	GGATCCCCCGGGCTGCAGGCGCCAATCGCAATGTTGAATG	CGAGGTCGACGGTATCGATATCAAGCGTAATCTGGAACATCGTATGGGTACGGTTGTGGTGTGATCTTAATTG	pMS024
***CBS1***	GGATCCCCCGGGCTGCAGGGAATCTCGACATGTTGAGGAC	CGAGGTCGACGGTATCGATACTAAGCGTAATCTGGAACATCGTATGGGTATGATTTACGCAAATGGTAATTAA	pMS025
***MPC1***	GGATCCCCCGGGCTGCAGGGATCAAAAAGAATGTCTCAACC	CGAGGTCGACGGTATCGATATTAAGCGTAATCTGGAACATCGTATGGGTACTGTTTACCAGTTTTTTC	pMS026
***MDM12***	GGATCCCCCGGGCTGCAGGCAACTAATCCAAATGTCTTTTG	CGAGGTCGACGGTATCGATATTAAGCGTAATCTGGAACATCGTATGGGTACTCATCACCATCGTTGAAATC	pMS023
***MDM34***	GGATCCCCCGGGCTGCAGGCAGCATCGTCATGTCATTTAG	CGAGGTCGACGGTATCGATATTAAGCGTAATCTGGAACATCGTATGGGTAATGATATGGTGGGGGGCTATC	pMS030
***UPS1***	GGATCCCCCGGGCTGCAGGCTTAAGAGTTGCAATGGTCCTTTTACAC	CGAGGTCGACGGTATCGATATCAAGCGTAATCTGGAACATCGTATGGGTAAAACTGAGGATTTCTCGC	pMS028
***PHB2***	GGATCCCCCGGGCTGCAGGATTTAGTGCTGATGAATAGATCACC	CGAGGTCGACGGTATCGATATTAAGCGTAATCTGGAACATCGTATGGGTATTTGCCCCTTCCATCGATTC	pMS027
***MRS4***	GGATCCCCCGGGCTGCAGGCAGTTAATATTATGAATACTTC	CGAGGTCGACGGTATCGATATCAAGCGTAATCTGGAACATCGTATGGGTAATTTTTCATTAAAAAATGCTTAG	pMS022
***MAS1***	GGATCCCCCGGGCTGCAGGGTGATCATACAATGTTTTCAAG	CGAGGTCGACGGTATCGATATCAAGCGTAATCTGGAACATCGTATGGGTATTGGTTCAGTTTTTCTTCG	pMS019
***MTC3***	GGATCCCCCGGGCTGCAGGTAAGGCAGAAGATGATGGGACG	CGAGGTCGACGGTATCGATATTAAGCGTAATCTGGAACATCGTATGGGTATACAAGATCCCTTAATTGTTC	pMS020
***PFD2***	GGATCCCCCGGGCTGCAGGTATATTACAGCTATGGAACAAAG	CGAGGTCGACGGTATCGATATTAAGCGTAATCTGGAACATCGTATGGGTAGTTTTTAACGACTTGAATC	pMS031

### Microscopy analysis and image processing

To visualize mitochondria, yeast cells were transformed with a plasmid that expressed mitochondrial-targeted GFP (pYX122) [59]. A 5 µl portion of the cell suspension was mixed with 45 µl of 0.5% agarose in 1 × phosphate-buffered saline (PBS) and mounted with a coverslip. Images were acquired with Zeiss Axio Imager M2 at × 100 magnification. Images were collected in *z*-stacks that consisted of 18 slices with a 0.588-µm depth in brightfield and 488 nm excitation with 200 and 2500 ms exposure times, respectively. The acquired images were further processed with ImageJ software. Only single, non-budding yeast cells were considered for the analysis. *Z*-stacked images were projected in sum intensity and subjected to consecutive adjustments (unsharp Mask: 5, range: 0.9; CLAHE: 30–50, if necessary; filter-median: 2.0). Pre-processed images were analyzed using the Mitochondrial Network Analysis (MINA) plugin tool without ridge detection. Analysis factors were reported previously [41] (high contrast: 75; low contrast: 5; line width: 1; minimum line length: 3). Mitochondrial morphology was expressed as “mean number of network points,” provided by the MINA tool. Three independent biological experiments were performed, and 29 individual yeast cells were analyzed for each strain. The statistical analysis was performed with Student’s *t* test (two-tailed, unpaired). In the case of unequal sample sizes, Mann–Whitney *U* non-parametric test was applied.

To visualize mitochondrial DNA (mtDNA) and the actin cytoskeleton, yeast cells were grown to the logarithmic growth phase and fixed with 3.7% formaldehyde in culture media for 60 min at room temperature. Samples were washed three times with 1 × PBS and stored at 4 °C overnight. The mtDNA was stained with DAPI (4’,6-diamidino-2-phenylindole; 1:200 dilution) for 15 min at room temperature with gentle shaking (200–300 rpm). Stained cells were washed three times with 1 × PBS and immediately prepared for microscopy as indicated above. Images were acquired using Zeiss Axio Imager M2 at × 100 magnification in *z*-stacks (18 slices at a 0.588 µm depth) using brightfield and excitation at 405 and 555 nm with 200, 1500, and 2000 ms exposure times, respectively. The acquired images were further processed in ImageJ using an unsharpened mask and the CLAHE tool as indicated above for the mitochondrial morphology analysis.

### Cell viability assay

To determine yeast viability, one OD_600_ unit from each culture was collected by centrifugation at 3000 × *g* for 5 min at room temperature. Pellets were resuspended in 1 ml of 1 × PBS. Propidium iodide (Sigma-Aldrich, catalog no. P4170, 1 mg/ml) was added to the cell suspension and incubated for 15 min at room temperature while protected from light. Propidium iodide stains only dead cells. Additionally, a negative control (no staining with propidium iodide) and a positive control were prepared for each sample. For the positive control, dead yeast cells were used, achieved by incubation at 80 °C for 10 min, which were stained with propidium iodide. Samples were diluted 1:10 in 1 × PBS and kept on ice until the analysis. All samples were analyzed by flow cytometry (BD FACS CALIBUR). Fluorescent signals were captured using BD CellQuest Pro software.

Data were processed using Flowing 2.5.1 software (Turku Bioscience Center) and are expressed as a percentage of dead cells. At least three independent biological repetitions were analyzed. The statistical analysis was performed using one-way analysis of variance (ANOVA) followed by the Tukey post hoc test.

### Analysis of membrane potential

One OD_600_ unit of yeast cells was incubated with 150 nM MitoTracker Red CMXRos (Thermo Fisher, catalog no. M7512). Cells were incubated for 30 min while protected from light. Samples were diluted 1:10 in 1 × PBS and kept on ice until the analysis. All samples were analyzed by flow cytometry (BD FACS CALIBUR). Fluorescent signals were captured using BD CellQuest Pro software.

Data were processed using Flowing 2.5.1 software (Turku Bioscience Center). The population of unstained cells was gated using the “dot plot” visualization tool. The population of stained cells was gated in the wildtype sample. The same gating was maintained for all samples within the same biological replicate. Fluorescent intensity, expressed as the geometric mean, was obtained from the “intensity plot” visualization tool. The statistical analysis was performed using Student’s *t* test (two-tailed, unpaired).

### Measurement of total ATP levels

Twenty OD_600_ units of yeast cells were harvested and washed once in lysis buffer (20 mM Tris acetate [pH 7.75] and 2 mM ethylenediaminetetraacetic acid [EDTA]). The yeast cell pellet was resuspended in lysis buffer and incubated at 90 °C for 5 min with agitation. The lysate was cleared by short centrifugation. The cleared lysate was analyzed using the ENLITEN ATP Assay kit (Promega) according to the manufacturer’s instructions. The statistical analysis was performed using one-way ANOVA followed by the Tukey post hoc test.

### Quantitative analysis of mtDNA copy number

Ten OD_600_ units of yeast cells were collected, and cells were incubated in lysis buffer (200 mM lithium acetate and 1% sodium dodecyl sulfate [SDS]) at 95 °C for 2 min. Samples were immersed in liquid nitrogen for 2 min and then incubated at 95 °C for 1 min. The freeze–thaw procedure was repeated once. DNA was extracted by adding chloroform and precipitated with 96% ethanol at − 20 °C overnight. DNA was resuspended in TE buffer (10 mM Tris–HCl and 1 mM EDTA, pH 8.0), and the concentration was measured using NanoDrop equipment. DNA (1 ng) was used for real-time quantitative PCR using SYBR Green. Specific primers for *COX1* (forward: 5′-CTA CAG ATA CAG CAT TTC CAA GA-3′; reverse: 5′-GTG CCT GAA TAG ATG ATA ATG GT-3′) [60], *COX3* (forward: 5′-ATT GAA GCT GTA CAA CCT ACC GAATT-3′; reverse: 5-′CCT GCG ATT AAG GCA TGATGA-3′), and *GAL4* (forward: 5′-TTT CTC CTG GCT CAG TAG GGC-3′; reverse: 5′-AGT TAC GAG AGG GTG GAC GGT-3′) [61] were used for amplification. Samples were analyzed in Roche LightCycler 480 (Basel, Switzerland) with the following parameters: 95 °C initial denaturation for 5 min, 40 cycles of 30 s at 95 °C, 20 s at 58 °C, and 20 s at 72 °C. Samples were analyzed in three technical repetitions. The mean CT value of each sample was normalized to mean CT value of *Gal4* and quantified by the standard 2^−∆∆Ct^ method [62]. Experiments were performed in three biological replicates. All values were statistically analyzed using Student’s *t* test (two-tailed, unpaired).

### RNA isolation and real-time RT-PCR

About 20 OD_600_ units of exponentially growing cells were collected by centrifugation and subjected to RNA isolation by the hot-phenol method as described previously [63]. RNA concentration was measured and an equal amount of isolated RNA (40 ng) was converted to cDNA by QuantiTect Reverse Transcription Kit (Qiagen, Hilden, Germany). Real-time RT-PCR was performed in 10 µl of reaction volume containing cDNA, RT-PCR Mix SYBR C (A&A Biotechnology, Gdynia, Poland) and specific primers. The name of genes and sequence of primers are listed in Table 3. For a given set of primers, negative controls and serial dilutions of cDNA, for generating a standard curve, were also loaded on a 384-well plate. The Plates were run on LightCycler 480 System (Roche, Basel, Switzerland) using an amplification program including 5 min (95 °C) followed by 40 cycles of 30 s (95 °C), 20 s (57 °C), and 20 s (72 °C). Melt curve profiles were analyzed following each experiment to ensure the specificity of amplification reactions. The relative expression of a tested gene to the geometrical mean of reference genes including *ACT1*, *ALG9*, and *TDH1* was calculated and used to generate a graph. The statistical analysis was performed using one-way ANOVA followed by the Tukey post hoc test.

**Table 3:** The sequence of primers used for quantitative RT-PCR in this study

Gene	Forward primer 5′3′	Reverse primer 5′3′	Reference
***ATP5***	TCCGAAAATAACAGACTGGG	GGATCCAATGGTTCAGCAC	This study
***ATP17***	GCCAGATACAAAGCCAAGTAC	CTCTTCCGCACCTTTATGATG	
***COX13***	GTCGCCCTACCAGCAATTG	TCGTAATCCCTTGGCCATTC	
***QCR8***	AACCTCATATGCTGTGTCTCC	ACCAATAAATTCCCGCAGG	
***SFC1***	TAACAACGCCATTCATGCG	ACCCTGATTAGTTGCTTGCC	
***PAM16***	CATGGACAAGATTAATAACAGG	GAGCCAGTTCCCATTTTAAC	
***PAM17***	TGGGCTGTAACGTTTCATGG	CACCAGAGGCTATTATCCCAG	
***TOM7***	AATGTAGCACATTATGGCTGG	CACTTGGTAACGGAGACAG	
***MRPL20***	ATCAGAATCCATCGCAACC	ACACTGCTTCGCTTTAGTG	
***MRPL25***	AGTTGGCACTGCGTTATGG	GGGCAAGAGAACACCTTTCATG	
***RPL21B***	AAAGCATGGTGCCGTTCAC	CCTTGGTAGAACTTGTGTGGC	
***RPL31B***	GAAGATGTCCGTCTAGCCCC	AGCAGAGGCAACCAAAACTG	
***RPS14A***	TCTAACGTTGTTCAAGCTCGT	ACCAGTAACTCTGGCGATGG	
***ACT1***	CATGTTCCCAGGTATTGCCG	GTCAAAGAAGCCAAGATAGA	[64]
***ALG9***	TCCATGATACAGGAGCAAGC	CTACCATCAGAACCGCATTC	
***TDH1***	GGTATGGCTTTCAGAGTCCCA	AGACAACGGCATCTTCGGTG	

### Total protein extraction and Western blot

Total protein extracts were prepared from 2.5 to 3 OD_600_ units of yeast cells. Extracts were prepared by precipitation with NaOH and trichloroacetic acid (TCA) according to Yaffe and Schatz [65]. Protein pellets were resuspended in Laemmli buffer that contained 50 mM dithiothreitol (DTT) and denatured at 65 °C for 15 min. Lysates that corresponded to 0.33 OD_600_ units were separated on 12.5% or 15% SDS–polyacrylamide gel electrophoresis (SDS-PAGE) gel. The protein gel was transferred to a polyvinylidene difluoride (PVDF) membrane (Millipore Sigma) using 1 × Transfer Buffer (20 mM Tris, 0.15 M glycine, and 20% methanol). The PVDF membrane was blocked with 5% nonfat milk in 1 × Tris-buffered saline for 2 h at room temperature and then incubated with specific primary antibodies overnight at 4 °C. Custom-raised antibodies against Por1, Cox4, Rpl17, Ilv2, Tom70, Atp5, Atp2, Rip1, and Mia40 were used at a dilution of 1:500 in 5% nonfat milk. Commercial antibodies were used against actin/Act1 (catalog no. MAB1501, Millipore, diluted 1:500), Pgk1 (catalog no. 459250, Invitrogen, diluted 1:2000), Rpt5 (catalog no. BML-PW8245), Hsp104 (catalog no. ADI-SPA-1040D), anti-FLAG tag (catalog no. F1804, Sigma, diluted 1:1000), anti-HA tag (catalog no. H9658, Sigma, diluted 1:5000), and anti-GFP (catalog no. 118144600001). Membranes were incubated with anti-rabbit immunoglobulin G (IgG) secondary antibodies (catalog no. A9169, Sigma, diluted 1:10,000) or anti-mouse secondary antibodies (catalog no. A4416, Sigma, diluted 1:5000) for 2 h at room temperature. Chemiluminescence protein signals were detected with X-ray films. Images were digitally processed using Photoshop software. Densitometry measurements were performed to quantify signals from the Western blot analysis using ImageJ software (National Institutes of Health, Bethesda, MD, USA). The statistical analysis was performed using Student’s *t* test (two-tailed, unpaired).

### Mitochondria isolation and cell fractionation

Yeast cells were grown in complete synthetic medium containing 3% glycerol and 0.05% glucose at 25 °C and shifted for 1 h to 53 °C. Mitochondria were isolated by differential centrifugation according to the standard method [66]. Three independent mitochondria isolations were performed and analyzed by blue native electrophoresis (as described below), denaturing electrophoresis and Western blot (as described above). For cell fractionation, yeast cells transformed with mtGFP-containing plasmid were grown in selective minimal medium containing 3% glycerol and 0.05% glucose at 25 °C. Forty-five OD_600_ units were fractionated by differential centrifugation [67].

### Analysis of respiratory chain complexes by Blue Native gel electrophoresis

Mitochondria were centrifuged at 20,000 × *g* for 5 min at 4 °C and the supernatant was discarded. Mitochondria pellets were resuspended in 75 µl of Digitonin buffer (20 mM Tris–HCl [pH 7.4], 50 mM NaCl, 10% glycerol, 0.1 mM EDTA [pH 8.0], 1% Digitonin, and 1 mM phenylmethylsulfonyl fluoride [PMSF]) corresponding to 1 µg/1 µl and incubated for 15 min on ice. Samples were centrifuged at 20,000 × *g* for 15 min at 4 °C. The resulting supernatant was mixed with 10 × loading dye (500 mM Ɛ-amino n-caproic acid, 100 mM Bis–Tris, and 5% Coomassie blue G-250) to a final volume of 15 µl for each condition and separated on 4–13% Blue Native PAGE gel at 4 °C. Next, the gel was transferred to a PVDF membrane (Millipore Sigma) using 1 × Transfer Buffer (20 mM Tris, 0.15 M glycine, and 5% methanol). Western blot was performed as described above.

### Analysis of insoluble protein fraction

Ten OD_600_ units of yeast cells were harvested, and pellets were resuspended in lysis buffer-B (30 mM Tris–HCl [pH 7.4], 150 mM NaCl, 20 mM KCl, 5 mM EDTA [pH 8.0], and 0.5 mM PMSF). Cells were disrupted with glass beads at 2200 rpm for 10 min at 4 °C. One percent Triton-X-100 (Serva Feinbiochemica, catalog no. 37238) was added to the lysate and incubated for 10 min at 4 °C. The supernatant was centrifuged at 4000 × *g* for 10 min at 4 °C. After centrifugation, the supernatant was divided, and 50% of the lysate was subjected to ultracentrifugation (Beckman Coulter, OptimaMax-130 K Ultracentrifuge, CA, USA) using an MLA-130 rotor (Beckman Coulter, catalog no. 06U882, CA, USA) at 125,000 × *g* for 1 h at 4 °C. The remaining 50% of the lysate was precipitated with 10% TCA (total protein fraction [T]). After ultracentrifugation, the supernatant fraction (soluble protein fraction [S]) was collected in a new Eppendorf tube, and the remaining pellet (insoluble protein fraction [P]) was carefully washed with lysis buffer-B, resuspended in urea sample buffer (6 M urea, 6% SDS, 125 mM Tris–HCl [pH 6.8], 0.01% Bromophenol Blue, and 50 mM DTT), and incubated at 37 °C for 8 min to ensure that the pellet was fully dissolved in the buffer. The supernatant fraction was precipitated with 10% TCA. TCA precipitates were collected by centrifugation at 20,000 × *g* for 15 min at 4 °C. After centrifugation, the pellets were washed with ice-cold acetone and resuspended in urea sample buffer. All samples were incubated at 37 °C for 15 min. An equal volume of all samples was separated on reducing SDS-PAGE, and Western blot was performed as described above.

### Immunoprecipitation

Fifty OD_600_ units of yeast cells were harvested, washed, and resuspended in lysis buffer (50 mM HEPES–KOH [pH 7.5], 100 mM NaCl, 1 mM EDTA, 5% glycerol, and 1% protease inhibitor cocktail [Roth, catalog no. 11873580001]). Cells were disrupted using glass beads at 2200 rpm for 30 min at 4 °C, and the lysate was clarified by spinning at 15,000 rpm at 4 °C for 15 min. Approximately 50 µl of Dynabeads goat anti-Mouse IgG (Invitrogen) was washed twice with washing buffer (0.5% bovine serum albumin in PBS). Dynabeads were resuspended in lysis buffer and coated with anti-HA antibody (Sigma, catalog no. H9658) for 4 h at 4 °C on a rotating mixer. The amount of beads and HA antibody used for each sample was comparable for lysates of cells grown at 25 °C and shifted to 37 °C. Thus, a similar amount of Pfd2-HA protein was captured in all samples. Ten percent of the cell lysate was saved (input), and the remaining lysate was loaded on 50 µl of antibody-coated Dynabeads. As a negative binding control, total cell lysate was mixed with naked Dynabeads (without HA antibody). The lysate-bead suspensions were incubated overnight (14–18 h) at 4 °C on a rotating mixer. After incubation, the beads were washed three times with lysis buffer, and the immunoprecipitated proteins were eluted by the addition of Laemmli buffer that contained 50 mM DTT to the beads (elution). An equivalent volume of Laemmli buffer that contained 50 mM DTT was added to the input fraction. Samples were heated at 65 °C for 15 min, and an equal volume of each sample was used to separate proteins by SDS-PAGE and analyzed by Western blot using specific antibodies.

### Analysis of genetic interactions and gene enrichment analysis

Genetic interactions were taken from the BioGRID 4.4 database (https://thebiogrid.org/). The functional enrichment analysis presented in Fig. 3B and Additional file 4 (Fig. S4B) was performed using g:Profiler (version: e107_eg54_p17_bf42210) with the g:SCS multiple testing correction method by applying a significance threshold of 0.05 [68]. The chart was plotted by http://www.bioinformatics.com.cn/srplot, an online platform for data visualization. Interaction network was generated using String online tool (version 11.5). Confidence level was set to 0.7.

### Sample preparation for proteomics analysis

Wildtype cells and Δ*pfd2* cells transformed with empty vector (pESC-URA) were grown in CSM-URA medium with 3% glycerol and 0.05% sucrose to logarithmic growth phase at 25 °C and harvested. Furthermore, independent cultures were grown in an identical manner but cultures were shifted from 25 °C to 37 °C for 4 h. Samples from 25 and 37 °C were processed independently. Four biological replicates were prepared per strain and growth temperature. Twenty-eight OD_600_ of yeast cells from each strain and condition were harvested, centrifuged to remove culture medium, frozen in liquid nitrogen, and stored at − 80 °C. Cell pellet was washed with sterile water and centrifuged at 4400 rpm for 5 min at 4 °C. Cell pellet was resuspended in 500 µl of 10% TCA and transferred to 2 ml tube with 200 µl of unwashed glass beads. PMSF was added to the final concentration of 2 mM. Cells were homogenized for 7 min at 4 °C vortex instrument (“Disruptor Genie”, Scientific Industries). Supernatant was collected into a 1.5-ml tube. Glass beads were washed with 250 µl of 10% TCA and vortexed gently. Supernatants were combined in a 1.5-ml tube and incubated on ice for 20 min. Supernatant was centrifuged at 14,000 rpm for 15 min at 4 °C. Pellet was washed with 500 µl of 100% ice-cold acetone and incubated for 10 min on ice. The sample was centrifuged at 14,000 rpm for 5 min at 4 °C. Pellet was resuspended in 8 M urea and incubated at 30 °C for 15 min to dissolve. The sample was centrifuged at 14,000 rpm for 2 min at 4 °C. The supernatant was transferred to a new 1.5-ml tube and protein concentration was determined using ROTI®Quant reagent (Carl Roth GmbH + Co. KG) in 96-well plate assay using bovine serum albumin (BSA) as a quantitation standard. For in-solution digestion, 50 µg of protein was mixed with ProteaseMAX™ Surfactant Trypsin Enhancer (Promega Corporation) in final concentration 0.05% and reduced using 5 mM DTT at 56 °C for 20 min and alkylated by 2-chloroacetamide (CAA) at a final concentration of 15 mM for 15 min at room temperature. Samples were digested by trypsin (enzyme to protein ratio 1:28) at 37 °C for 16 h (400 rpm). Proteolysis was stopped by adding TFA to final concentration 0.5%. The digest was snap-frozen and stored at − 80 °C. Peptide mixtures were desalted with the use of AttractSPE™ Disks Bio-C18 (Affinisep, cat. no. SPE-Disks-Bio-C18-100.T1.47.20) using a previously reported stage-tip protocol [69] and dried in a centrifugal vacuum concentrator at 40 °C. Prior to LC–MS/MS measurement, the samples were resuspended in 0.1% TFA, 2% acetonitrile in water.

### LC–MS/MS analysis

Chromatographic separation was performed on an Easy-Spray Acclaim™ PepMap™ column 50 cm length × 75 µm inner diameter (Thermo Fisher Scientific) at 55 °C by applying 90 min acetonitrile gradients in 0.1% aqueous formic acid at a flow rate of 300 nl/min. An UltiMate™ 3000 nano-LC system was coupled to a Q Exactive HF-X mass spectrometer via an Easy-Spray source (all Thermo Fisher Scientific). The Q Exactive™ HF-X was operated in data-dependent mode with survey scans acquired at a resolution of 120,000 at m/z 200. Up to 12 of the most abundant isotope patterns with charges 2–5 from the survey scan were selected with an isolation window of 1.3 m/z and fragmented by higher-energy collision dissociation (HCD) with normalized collision energies of 27, while the dynamic exclusion was set to 30 s. The maximum ion injection times for the survey scan and the MS/MS scans (acquired with a resolution of 15,000 at m/z 200) were 45 and 96 ms, respectively. The ion target value for MS was set to 3 × 1e6 and for MS/MS to 1e5, and the intensity threshold for MS/MS was set to 1e4.

### Processing of proteomics data, Visualization and statistical analysis

Sixteen raw files were loaded in MaxQuant version 2.2.0.0 [70]. Oxidation of methionine, protein N-terminal acetylation, and deamidation of asparagine (N) and glutamine (Q) were specified as variable modifications. Cysteine carbamidomethylation was specified as a fixed modification. Trypsin/P was selected as protease allowing for up to two missed cleavages. Label-free quantification (LFQ) algorithm was used. Reviewed UniProtKB sequences of *Saccharomyces cerevisiae* (downloaded at 17th February 2023, includes 6727 protein sequences) were digested in silico and used by the Andromeda search engine. The false discovery rates (FDR) at the peptide spectrum match level (PSM) and at the protein level were set on 0.01.

The result of MaxQuant search generated in proteinGroups.txt output table were processed further using Perseus ver. 2.0.6.0 [71]. The dataset with LFQ intensities as main columns was filtered to exclude commonly occurring contaminants, proteins identified by site or matched to the reverse database. Protein groups that were identified by less than 2 razor + unique peptides were removed. Upon inspection of the Gaussian distribution of all samples, the biological replicate 3 of sample Δpfd2_37°C and biological replicate 4 of sample WT_37°C was removed from further analysis because of larger number of missing values. LFQ intensities were log2-transformed and rows were filtered based on valid values. Proteins with at least 50% of valid values within all four conditions were maintained. All remaining missing values were imputed from normal distribution (width = 0.3, down shift = 1.8, mode: separately for each column). The Gaussian distribution of log2-transformed LFQ intensities after imputation was confirmed for each sample by histogram analysis. Using build-in statistical analysis in Perseus v2.0.9.0, *p*-values between two groups (Δpfd2_25°C vs. WT_25°C; Δpfd2_37°C vs. WT_37°C; Δpfd2_37°C vs. Δpfd2_25°C; WT_37°C vs. WT_25°C) were determined by two-sided Welch’s *t*-test (permutation-based FDR with 250 randomizations = 0.05, S0 = 0). Corrected Welch’s *t*-test *p*-values are presented as adj. *p*-values in Additional file 10. Protein groups exhibiting an adj. *p*-value < 0.05 and a minimum fold change ± 1.3 were classified as up- or downregulated. Source data for the proteomics analysis are presented in Additional file 10 (Table S1).

### Gene Ontology annotation for proteomics data

Gene Ontology (GO) annotations for cellular components (CC), molecular function (MF), and biological process (BP) of yeast proteome were retrieved on 11.10.2022 from UniProtKB for strain ATCC 204508 / S288c, Proteome ID UP000002311. To obtain information about the cellular localization of proteins, GO CC annotations were consolidated into nine main annotations according to parent–child terms, using as the highest degree parent terms corresponding to Cytosol, Nucleus, Endoplasmic reticulum, Golgi apparatus, Mitochondrion, Vacuole, Peroxisome, Lysosome, and Ribosome. Terms annotated with “Cytosol” and “Ribosome” were named “Cytosolic Ribosome”, while terms named “Mitochondrion” and “Ribosome” were named “Mitochondrial Ribosome”. Terms annotated as “Cytosol” and “Nucleus” were assigned to a group called “Cytosol/ Nucleus”. Proteins annotated as localized in other subcellular compartments were assigned to a group “Other”. For proteasomal localization, GO CC terms were searched for the words “proteasome” and “proteasomal”. To assess if a protein function as a chaperone, GO BP and GO MF terms were searched for words “folding,” “refolding,” and “chaperone”. The annotation search was performed with Python version 3.9 and Microsoft Excel 2016.

### GO enrichment analysis and protein association network analysis related to proteomics data

Analysis of GO enrichment was performed using the TopGo package in R version 4.1.0 with annotations retrieved on 23.05.2023 from Ensemble for the organism *S. cerevisiae*. Proteins significantly upregulated (fold change > 1.3, FDR < 0.05) or downregulated (fold change < -1.3, FDR < 0.05) in each condition were subjected to the GO enrichment analysis for biological process (BP), molecular function (MF), and cellular components (CC). As background, all proteins detected in the mass spectrometry experiment were used. GO terms with at least 5 genes per term were used. The algorithm weight01 with Fisher’s statistics was used to calculate the *p*-value. The top 200 terms were selected and terms were filtered for fold enrichment > 1.5, *p*-value < 0.05, and a minimum number of significant genes = 2. Benjamini-Hochberg (BH) correction was used to adjust for multiple testing. The fold enrichment was calculated as the number of significant genes found in a GO term divided by the number of expected genes taken from the submitted background.

Generation of the protein association network was performed with Cytoscape 3.9.1. [72] with a plugin StringApp 1.7.1. [73]. The confidence for interaction was set to 0.7. The thickness of the edges indicated the strength of evidence of the interaction in the literature. For the display, the structure previews were disabled and a prefuse force-directed openCL layout was used. Proteins were colored according to the mitochondrial and ribosomal localization, presence in the proteasome, and involvement in the protein folding process.

### Supplementary Information

**Additional file 1:** **(Fig. S1.; Related to Fig. 1).** Growth tests of cells deleted of single prefoldin subunits. A-D Ten-fold dilutions of wildtype cells and cells that lacked genes that encode prefoldin subunits were spotted on agar plates that were supplemented as indicated. Cells were grown at 28°C for 2-3 days. A, B Experiments testing oxidative stress and salt stress were performed in two biological repetitions. C Experiments testing different means of osmotic stress were performed once. D Experiments testing UV stress were performed in three biological repetitions. E Single prefoldin deletions are sensitive to high temperature. Ten-fold dilutions of wildtype cells and cells that lacked genes that encode prefoldin subunits were spotted on complete synthetic medium plates that contained glucose or glycerol. Cells were grown at the indicated temperatures for 3 days. Experiments were performed in two biological repetitions. F, G Yeast cells were grown at 25°C in complete synthetic medium that contained glycerol until the logarithmic growth phase (F) and shifted to 53°C for 1 h (G). Cell viability was assessed by propidium iodide staining and analyzed by flow cytometry. The data are expressed as the mean ± SEM. *n* = 3. ***p < 0.001, *p< 0.05. WT, wild type.

**Additional file 2: (Fig. S2.; Related to Fig. 1).** Growth test of cells upon mitochondrial stress. A Ten-fold dilutions of yeast cells of the indicated strains were spotted on solid agar plates with full medium that contained galactose supplemented with antimycin A or an equal volume of ethanol (control), which was used as the solvent. Cells were grown at 28°C for 2 days. Experiments were performed in two biological repetitions. B Quantification of drop tests. The data are expressed as the mean± SD. *n* = 2. C Yeast cells were grown in full medium that contained glycerol at 28°C. Total protein extract was separated on SDS-PAGE and analyzed by Western blot using specific antibodies. D Quantification of relative protein levels. The data are expressed as the mean ± SEM. *n* = 3. E Cells were grown in complete synthetic medium containing glycerol at 25°C. Protein extracts were separated by SDS-PAGE and analyzed by Western blot against specific antibodies. F Quantification of total GFP levels. The data are expressed as the mean ± SEM. *n* = 3. ns, not significant.  G Total cell extracts were fractionated. T, total protein extract; S, post-mitochondrial supernatant; M, mitochondrial fraction. Equal volume of all fractions was loaded on SDS-PAGE and analyzed by Western blot against specific antibodies. H Quantification of GFP levels in the post-mitochondrial supernatant. The data are expressed as the mean ± SEM. *n* = 3. ns, not significant. I Five-fold dilutions of cells were spotted on selective medium plates that contained glucose or galactose (induction of expression of *PFD2-Flag* and *Flag-PFD5*). Plates were supplemented with antimycin A or an equal volume of ethanol (solvent control). Cells were grown at 28°C for 6 days. The experiments were performed in at least two biological repetitions. WT, wild type. Uncropped blots for panel C, E, and G are presented as source data in the Additional file13.

**Additional file 3:** **(Fig. S3; Related to Fig. 2). **Characterization of mitochondrial function upon loss of single prefoldin subunits. A, D-F Yeast cells were grown in complete synthetic medium that contained glycerol. A Wildtype cells were treated with 5 µM oligomycin for 1.5 h prior harvesting (Oligo). Cells were stained with MitoTracker Red CMXRos and analyzed by flow cytometry. The data are expressed as the geometric mean ± SEM of stained cells. *n* = 5. ns, not significant. B, C Total ATP levels were measured in whole cell lysate. The data are expressed as mean ± SEM fold changes relative to wild type. *n* = 3. *p < 0.05. ns, not significant. D Formaldehyde-fixed cells were incubated with DAPI. The figure shows consecutive Z-stack images for each strain. Experiments were performed in two biological repetitions. At least nine cells per strain were analyzed. Scale bar = 5 µm. E, F Analysis of mitochondrial DNA copy number for the *COX1* and *COX3* genes. Values were normalized to the DNA copy number of a nuclear gene, *GAL4*. The data are expressed as the mean ± SEM. *n* = 3. *p< 0.05; ns, not significant; WT, wild type.

**Additional file 4: (Fig. S4.; Related to Fig. 3).** Deletions of prefoldin subunits have distinct negative genetic interaction partners. A UpSet plot illustrating the number of genes that when deleted result in a negative genetic interaction only with one prefoldin subunit (unique negative genetic interaction). B Gene ontology analysis of overrepresented terms within unique negative genetic interaction partners. No enrichment of GO terms was identified for negative genetic interactions with *Δpfd1*.

**Additional file 5: (Fig. S5.; Related to Fig. 3).** Overexpression of mitochondrial genes in Δ*pfd2*. A String network of mitochondrial genes that when deleted result in  negative genetic interaction with *Δpfd2*. Marked in red are genes that grouped into the GO term “Mitochondrial membrane” (GO: 0031966). B Wildtype cells were transformed with an empty vector. Δ*pfd2* cells were transformed with an empty vector or a plasmid that harbored mitochondrial genes that were identified as negative genetic interactors with Δ*pfd2. *The genes expression was under control of the constitutive *TEF1* promoter. Transformed strains were grown on selective minimal medium that contained glycerol at 25°C to the logarithmic growth phase and shifted to 42°C for 6 h. Cell viability was assessed by propidium iodide staining and analyzed by flow cytometry. The data are presented as the mean ± SEM. *n* = 4. *p < 0.05. C Wildtype cells and cells that lacked *PFD2* were transformed with Mas1-HA. Transformed cells were grown on selective minimal medium that contained glycerol at 25°C to the logarithmic growth phase and shifted to 42°C for 6 h. Insoluble proteins were obtained from the whole cell lysate by ultracentrifugation. Fractions (T, total cell extract 42°C; P, pellet; S, supernatant; T’, total cell extract 25°C) were separated on SDS-PAGE and analyzed by Western blot using specific antibodies. Uncropped blots are presented as source data in the Additional file 13. D Mas1-HA protein levels were quantified, and levels were normalized to Rpl17 protein levels. the representative blot shows two different yeast transformants (Col1 and Col2) for wildtype and Δ*pfd2 *strains. The data are expressed as the mean ± SEM. *n* = 4 (for WT + *MAS1*-HA),*n* = 3 (for Δ*pfd2* + *MAS1*-HA). WT, wild type.

**Additional file 6: (Fig. S6; Related to Fig. 4).** Heat shock of Δ*pfd2* cells does not result in significant changes on the proteome level compared to wildtype cells. A Cells were grown in complete minimal medium to logarithmic growth phase at 25°C. The mRNA levels of the indicated genes were analyzed by quantitative real time PCR. The data are expressed as relative levels compared with the geometric mean of the house keeping genes *ACT1*, *TDH1*, and *ALG9*. *n* = 3. ***p < 0.001, **p< 0.01, *p < 0.05. ns, not significant. B-F Comparison of changes in proteome of Δ*pfd2* compared with wildtype cells at 37°C growth temperature. Volcano plot showing all identified proteins that localize to mitochondria (light pink circles in B), mitochondrial ribosome (dark pink circle in C), cytosolic ribosome (green circle in D), chaperones and protein folding (yellow circle in E), and proteasome (brown circle in F). WT, wild type.

**Additional file 7: (Fig. S7; Related to Fig. 4).** Comparison of heat shock response in wildtype cells and Δ*pfd2* cells. A, B Proteomes of wildtype (A) or Δ*pfd2* (B) cells grown at 25°C and shifted to 37°C for 4h were compared. Volcano plots showing proteins with significantly increased (blue circles) and decreased (red circles) protein abundance in wildtype cells 37°C vs. 25°C or Δ*pfd2* cells 37°C vs. 25°C. Adj. *P*-value of <0.05 (FDR = 0.05) and fold change of 1.3 was considered as statistical significant change. Non-significant proteins are shown in gray circles. C-L Volcano plots showing all identified proteins that localize to mitochondria (light pink circles in C, H), mitochondrial ribosome (dark pink circle in D, I), cytosolic ribosome (green circle in E, J), chaperones and protein folding (yellow circle in F, K), and proteasome (brown circle in G, L). C-G Comparison of changes in proteome of wildtype cells grown at 37°C with wildtype cells grown at 25°C. H-L Comparison of changes in proteome of Δ*pfd2* cells grown at 37°C with Δ*pfd2* cells grown at 25°C. WT, wild type.

**Additional file 8: (Fig. S8; Related to Fig. 4).** Cellular responses of wildtype cells upon heat shock. A Gene ontology enrichment of proteins with significantly downregulated (upper panel) or upregulated (lower panel) protein abundance in wildtype cells grown at 37°C compared with wildtype grown at 25°C. Values next to the right side of the bars indicate the numbers of proteins with the certain GO term. B Interaction network of all proteins significantly downregulated. In pink are proteins localized to mitochondria and in green are proteins of the cytosolic ribosome marked. Other proteins are shown in gray. C Interaction network of all proteins significantly upregulated. In pink are proteins with mitochondrial localization, in yellow are proteins identified as chaperones. Other proteins are shown in gray. WT, wild type.

**Additional file 9: (Fig. S9; Related to Fig. 4).** Cellular responses of Δ*pfd2* cells upon heat shock. A Gene ontology enrichment of proteins with significantly downregulated (upper panel) or upregulated (lower panel) protein abundance in Δ*pfd2* cells grown at 37°C compared with Δ*pfd2* grown at 25°C. Values next to the right side of the bars indicate the numbers of proteins with the certain GO term. B Interaction network of all proteins significantly downregulated. In pink are proteins localized to mitochondria. Other proteins are shown in gray. C Interaction network of all proteins significantly upregulated. In pink are proteins with mitochondrial localization, in yellow are proteins identified as chaperones, and in brown a protein of the proteasome is shown. Other proteins are shown in gray.

**Additional file 10: (Table S1).** Source file of mass spectrometry data comparing whole yeast proteome of Δ*pfd2* and wildtype cells grown at 25°C and shifted for 4h to 37°C. Sheet 1 (“Non-imputed data”) contain non-imputed data. LFQ intensities marked with 0 refer to unidentified values. Sheet 2 (“Imputed data+statistics”) contain data were unidentified values were imputed using normal distribution option in Perseus [71]. Sheet 3 (“Data_For_Volcano”) contain source data cellular localization and classification of proteins into the selected categories “Proteasome”, “Chaperones/ Protein folding”, “Mitochondrion”,“MitoRibo” (mitochondrial ribosome), “CytoRibo” (cytosolic ribosome).

**Additional file 11: (Table S2). **Gene ontology enrichment of proteins with significantly upregulated or downregulated protein abundance comparing the following groups:  Δ*pfd2*_25°C vs. WT_25°C, Δ*pfd2*_37°C vs. Δ*pfd2*_25°C,  WT_37°C vs. WT_25°C.

**Additional file 12: (Table S3). **Source data underlying the numerical data presented in the manuscript and list of genetic interactors obtained from BioGRID database used to generate Figure 3A and Additional file 4 (Fig. S4A) including references to original publications which reported the genetic interaction.

**Additional file 13. **Source data presenting uncropped western blots.

## Data Availability

The authors confirm that all relevant data are included in the main manuscript and Additional files provided with the manuscript. The mass spectrometry proteomics data have been deposited to the ProteomeXchange Consortium via the PRIDE [74] partner repository with the dataset identifier PXD040390. The materials that were generated within this work are available upon request from the corresponding author (U.T., utopf@ibb.waw.pl).

## Abbreviations

TRiC: Tailless complex polypeptide 1 ring complex
CCT: Chaperonin containing tailless complex 1
ATP: Adenosine triphosphate
PFD or PFDN: Prefoldin
OD_600_: Optical density at 600 nm
Hsp: Heat shock protein
TOM: Translocase of the outer membrane in mitochondria
UV: Ultraviolet
GFP: Green fluorescent protein
PCR: Polymerase chain reaction
GO: Gene ontology
EV: Empty vector
HA: Hemagglutinin
CSM: Complete synthetic medium
mtGFP: Mitochondrial-targeted GFP
PBS: Phosphate-buffered saline
MINA: Mitochondrial Network Analysis plugin
mtDNA: Mitochondrial DNA
DAPI: 4’,6-Diamidino-2-phenylindole
ANOVA: Analysis of variance
EDTA: Ethylenediaminetetraacetic acid
SDS: Sodium dodecyl sulfate
TCA: Trichloroacetic acid
DTT: Dithiothreitol
SDS-PAGE: SDS–polyacrylamide gel electrophoresis
PVDF: Polyvinylidene difluoride
IgG: Immunoglobulin G
PMSF: Phenylmethylsulfonyl fluoride
CAA: 2-Chloroacetamide
TFA: Trifluoroacetic acid
HCD: Higher-energy collision dissociation
LFQ: Label-free quantification
FDR: False discovery rate
PSM: Peptide spectrum match
CC: Cellular components
MF: Molecular function
BP: Biological process
Oligo: Oligomycin
MitoRibo: Mitochondrial ribosome
CytoRibo: Cytosolic ribosome
LC–MS/MS: Liquid chromatography tandem mass spectrometry
