# Supplementary material for: Prefoldin 2 contributes to mitochondrial morphology and function
Source: BMC Biol. 2023 Sep 12;21:193. doi: 10.1186/s12915-023-01695-y (PMC10496292; doi:10.1186/s12915-023-01695-y)
Supplement: Supplementary file 1 — Additional file 1: (Fig. S1.; Related to Fig. 1). Growth tests of cells deleted of single prefoldin subunits. A-D Ten-fold dilutions of wildtype cells and cells that lacked genes that encode prefoldin subunits were spotted on agar plates that were supplemented as indicated. Cells were grown at 28°C for 2-3 days. A, B Experiments testing oxidative stress and salt stress were performed in two biological repetitions. C Experiments testing different means of osmotic stress were performed once. D Experiments testing UV stress were performed in three biological repetitions. E Single prefoldin deletions are sensitive to high temperature. Ten-fold dilutions of wildtype cells and cells that lacked genes that encode prefoldin subunits were spotted on complete synthetic medium plates that contained glucose or glycerol. Cells were grown at the indicated temperatures for 3 days. Experiments were performed in two biological repetitions. F, G Yeast cells were grown at 25°C in complete synthetic medium that contained glycerol until the logarithmic growth phase (F) and shifted to 53°C for 1 h (G). Cell viability was assessed by propidium iodide staining and analyzed by flow cytometry. The data are expressed as the mean ± SEM. n = 3. ***p < 0.001, *p< 0.05. WT, wild type. [file 12915_2023_1695_MOESM1_ESM.pdf]

Additional file 1

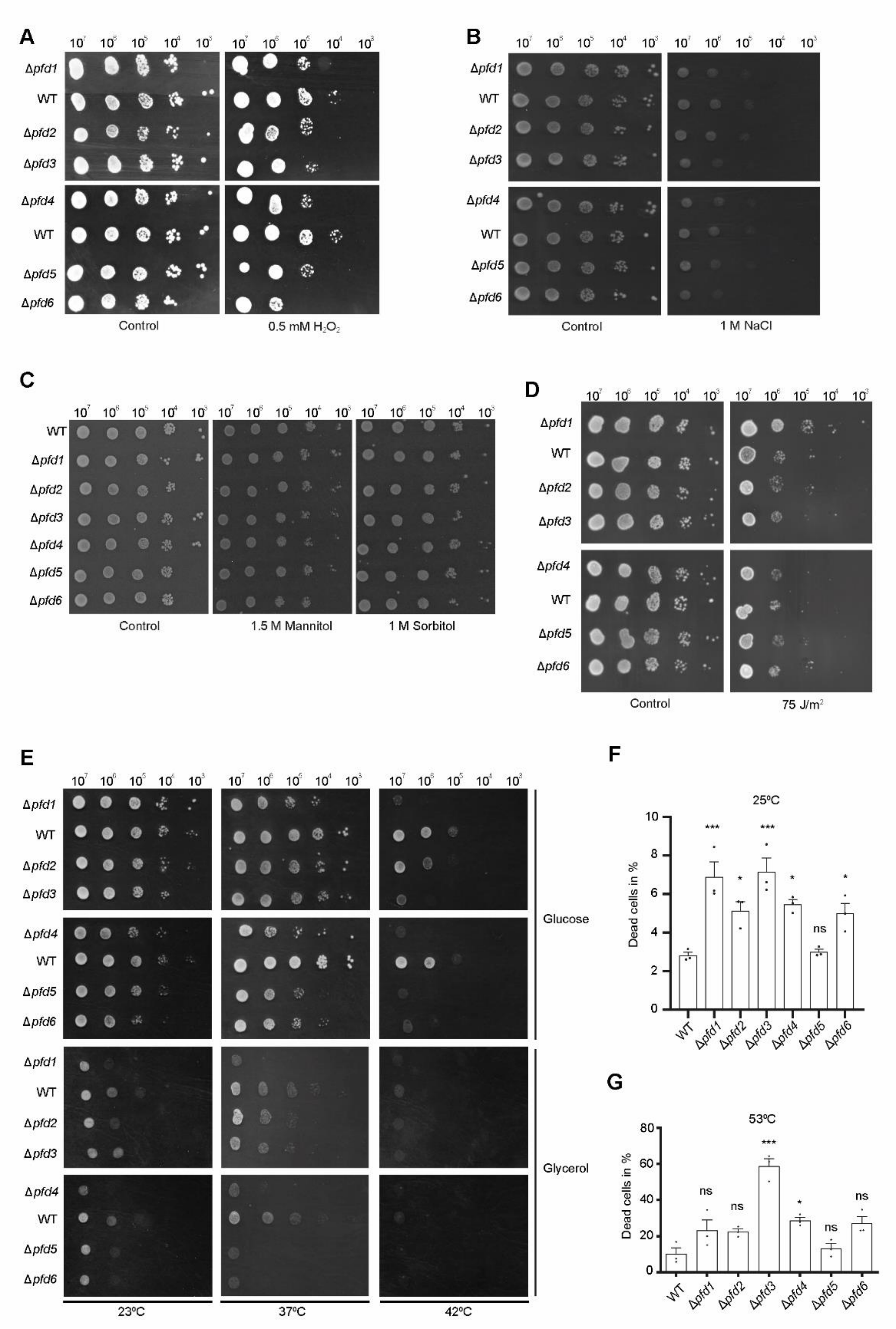

Fig. S1.; Related to Fig. 1. Growth tests of cells deleted of single prefoldin subunits. **A-D** Ten-fold dilutions of wildtype cells and cells that lacked genes that encode prefoldin subunits were spotted on agar plates that were supplemented as indicated. Cells were grown at 28°C for 2-3 days. **A, B** Experiments testing oxidative stress and salt stress were performed in two biological repetitions. **C** Experiments testing different means of osmotic stress were performed once. **D** Experiments testing UV-stress were performed in three biological repetitions. **E** Single prefoldin deletions are sensitive to high temperature. Ten-fold dilutions of wildtype cells and cells that lacked genes that encode prefoldin subunits were spotted on complete synthetic medium plates that contained glucose or glycerol. Cells were grown at the indicated temperatures for 3 days. Experiments were performed in two biological repetitions. **F, G** Yeast cells were grown at 25°C in complete synthetic medium that contained glycerol until the logarithmic growth phase (F) and shifted to 53°C for 1 h (G). Cell viability was assessed by propidium iodide staining and analyzed by flow cytometry. The data are expressed as the mean ± SEM. $n = 3$. ***$p < 0.001$, *$p < 0.05$. WT, wild type.
