# Supplementary material for: Prefoldin 2 contributes to mitochondrial morphology and function
Source: BMC Biol. 2023 Sep 12;21:193. doi: 10.1186/s12915-023-01695-y (PMC10496292; doi:10.1186/s12915-023-01695-y)
Supplement: Supplementary file 2 — Additional file 2: (Fig. S2.; Related to Fig. 1). Growth test of cells upon mitochondrial stress. A Ten-fold dilutions of yeast cells of the indicated strains were spotted on solid agar plates with full medium that contained galactose supplemented with antimycin A or an equal volume of ethanol (control), which was used as the solvent. Cells were grown at 28°C for 2 days. Experiments were performed in two biological repetitions. B Quantification of drop tests. The data are expressed as the mean± SD. n = 2. C Yeast cells were grown in full medium that contained glycerol at 28°C. Total protein extract was separated on SDS-PAGE and analyzed by Western blot using specific antibodies. D Quantification of relative protein levels. The data are expressed as the mean ± SEM. n = 3. E Cells were grown in complete synthetic medium containing glycerol at 25°C. Protein extracts were separated by SDS-PAGE and analyzed by Western blot against specific antibodies. F Quantification of total GFP levels. The data are expressed as the mean ± SEM. n = 3. ns, not significant. G Total cell extracts were fractionated. T, total protein extract; S, post-mitochondrial supernatant; M, mitochondrial fraction. Equal volume of all fractions was loaded on SDS-PAGE and analyzed by Western blot against specific antibodies. H Quantification of GFP levels in the post-mitochondrial supernatant. The data are expressed as the mean ± SEM. n = 3. ns, not significant. I Five-fold dilutions of cells were spotted on selective medium plates that contained glucose or galactose (induction of expression of PFD2-Flag and Flag-PFD5). Plates were supplemented with antimycin A or an equal volume of ethanol (solvent control). Cells were grown at 28°C for 6 days. The experiments were performed in at least two biological repetitions. WT, wild type. Uncropped blots for panel C, E, and G are presented as source data in the Additional file13. [file 12915_2023_1695_MOESM2_ESM.pdf]

Additional file 2

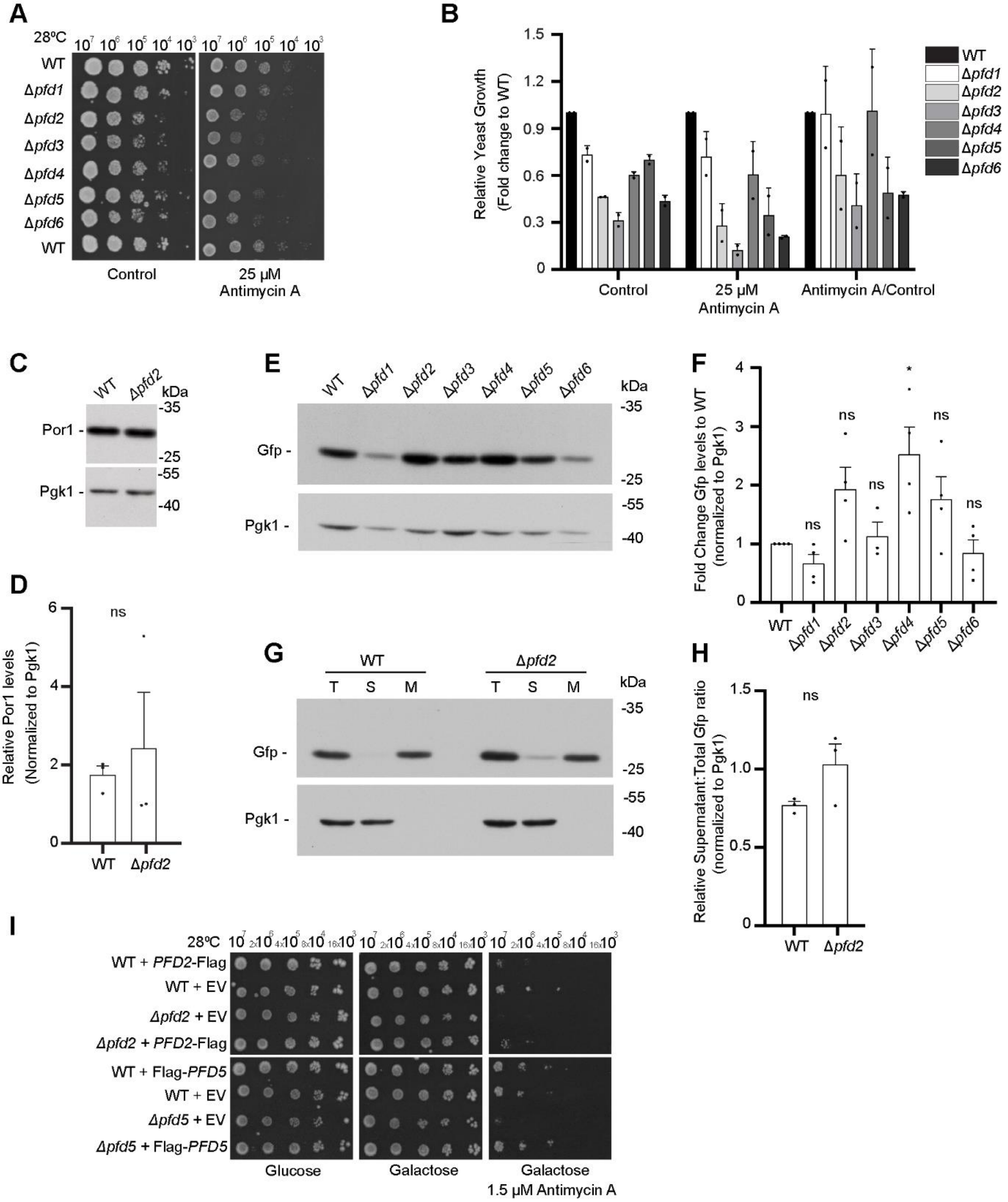

Fig. S2.; Related to Fig. 1. Growth test of cells upon mitochondrial stress. **A** Ten-fold dilutions of yeast cells of the indicated strains were spotted on solid agar plates with full medium that contained galactose supplemented with antimycin A or an equal volume of ethanol (control), which was used as the solvent. Cells were grown at 28°C for 2 days. Experiments were performed in two biological repetitions. **B** Quantification of drop tests. The data are expressed as the mean ± SD. *n* = 2. **C** Yeast cells were grown in full medium that contained glycerol at 28°C. Total protein extract was separated on SDS-PAGE and analyzed by Western blot using specific antibodies. **D** Quantification of relative protein levels. The data are expressed as the mean ± SEM. *n* = 3. **E** Cells were grown in complete synthetic medium containing glycerol at 25°C. Protein extracts were separated by SDS-PAGE and analysed by Western blot against specific antibodies. **F** Quantification of total GFP levels. The data are expressed as the mean ± SEM. *n* = 3. ns, not significant. **G** Total cell extracts were fractionated. T, total protein extract; S, post-mitochondrial supernatant; M, mitochondrial fraction. Equal volume of all fractions was loaded on SDS-PAGE and analysed by Western blot against specific antibodies. **H** Quantification of GFP levels in the post-mitochondrial supernatant. The data are expressed as the mean ± SEM. *n* = 3. ns, not significant. **I** Five-fold dilutions of cells were spotted on selective medium plates that contained glucose or galactose (induction of expression of *PFD2-Flag* and *Flag-PFD5*). Plates were supplemented with antimycin A or an equal volume of ethanol (solvent control). Cells were grown at 28°C for 6 days. The experiments were performed in at least two biological repetitions. WT, wild type. Uncropped blots for panel C, E, and G are presented as source data in the Additional file 13.
