# Supplementary material for: Prefoldin 2 contributes to mitochondrial morphology and function
Source: BMC Biol. 2023 Sep 12;21:193. doi: 10.1186/s12915-023-01695-y (PMC10496292; doi:10.1186/s12915-023-01695-y)
Supplement: Supplementary file 3 — Additional file 3: (Fig. S3; Related to Fig. 2). Characterization of mitochondrial function upon loss of single prefoldin subunits. A, D-F Yeast cells were grown in complete synthetic medium that contained glycerol. A Wildtype cells were treated with 5 µM oligomycin for 1.5 h prior harvesting (Oligo). Cells were stained with MitoTracker Red CMXRos and analyzed by flow cytometry. The data are expressed as the geometric mean ± SEM of stained cells. n = 5. ns, not significant. B, C Total ATP levels were measured in whole cell lysate. The data are expressed as mean ± SEM fold changes relative to wild type. n = 3. *p < 0.05. ns, not significant. D Formaldehyde-fixed cells were incubated with DAPI. The figure shows consecutive Z-stack images for each strain. Experiments were performed in two biological repetitions. At least nine cells per strain were analyzed. Scale bar = 5 µm. E, F Analysis of mitochondrial DNA copy number for the COX1 and COX3 genes. Values were normalized to the DNA copy number of a nuclear gene, GAL4. The data are expressed as the mean ± SEM. n = 3. *p< 0.05; ns, not significant; WT, wild type. [file 12915_2023_1695_MOESM3_ESM.pdf]

Additional file 3

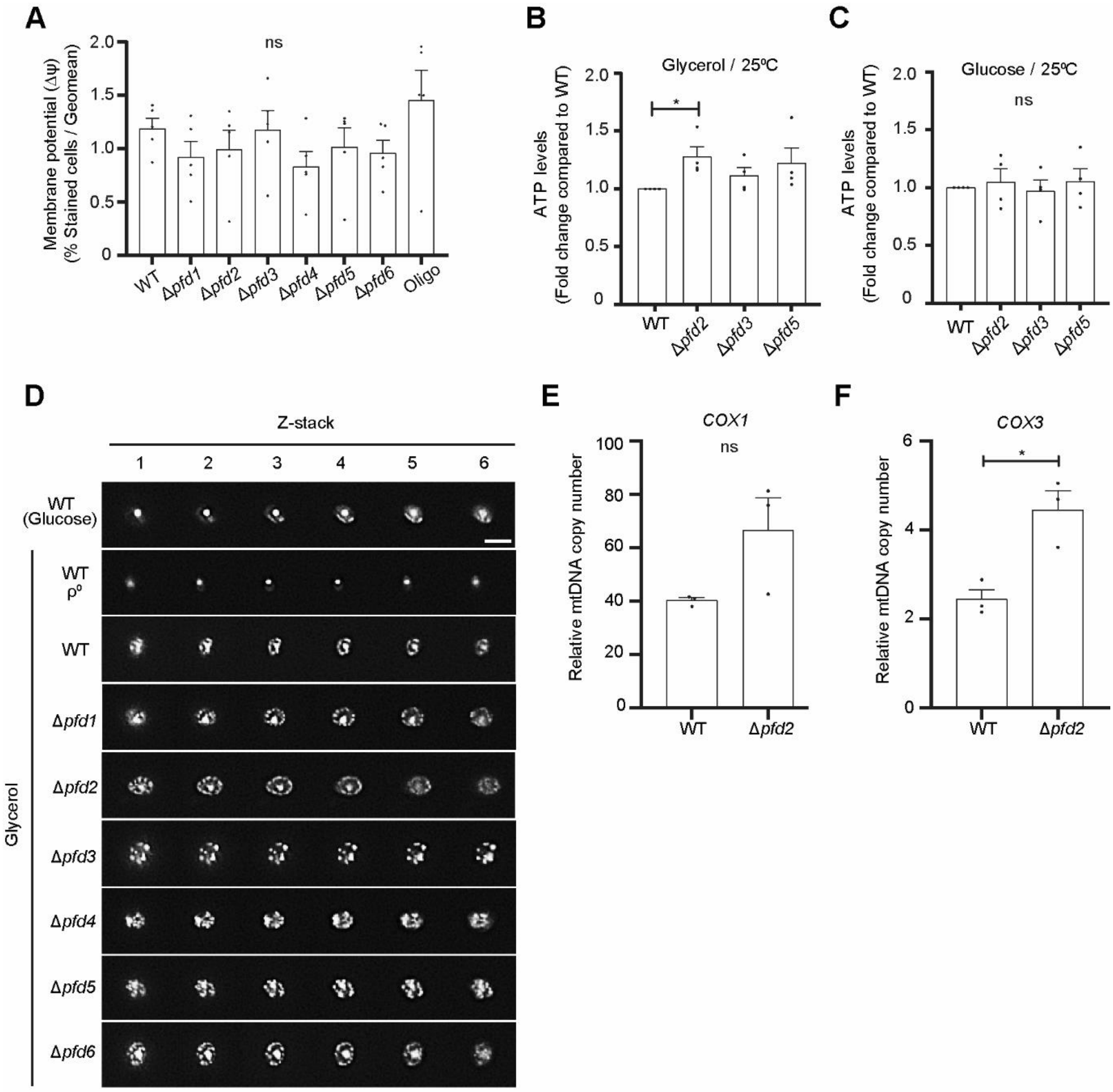

Fig. S3; Related to Fig. 2. Characterization of mitochondrial function upon loss of single prefoldin subunits. **A**, **D-F** Yeast cells were grown in complete synthetic medium that contained glycerol. **A** Wildtype cells were treated with 5 µM oligomycin for 1.5 h prior harvesting (Oligo). Cells were stained with MitoTracker Red CMXRos and analyzed by flow cytometry. The data are expressed as the geometric mean ± SEM of stained cells. *n* = 5. ns, not significant. **B, C** Total ATP levels were measured in whole cell lysate. The data are expressed as mean ± SEM

fold changes relative to wild type. $n = 3$. *$p < 0.05$. ns, not significant. **D** Formaldehyde-fixed cells were incubated with DAPI. The figure shows consecutive Z-stack images for each strain. Experiments were performed in two biological repetitions. At least nine cells per strain were analysed. Scale bar = 5 µm. **E, F** Analysis of mitochondrial DNA copy number for the *COX1* and *COX3* genes. Values were normalized to the DNA copy number of a nuclear gene, *GAL4*. The data are expressed as the mean ± SEM. $n = 3$. *$p < 0.05$; ns, not significant; WT, wild type.
