# Supplementary material for: Prefoldin 2 contributes to mitochondrial morphology and function
Source: BMC Biol. 2023 Sep 12;21:193. doi: 10.1186/s12915-023-01695-y (PMC10496292; doi:10.1186/s12915-023-01695-y)
Supplement: Supplementary file 4 — Additional file 4: (Fig. S4.; Related to Fig. 3). Deletions of prefoldin subunits have distinct negative genetic interaction partners. A UpSet plot illustrating the number of genes that when deleted result in a negative genetic interaction only with one prefoldin subunit (unique negative genetic interaction). B Gene ontology analysis of overrepresented terms within unique negative genetic interaction partners. No enrichment of GO terms was identified for negative genetic interactions with Δpfd1. [file 12915_2023_1695_MOESM4_ESM.pdf]

Additional file 4

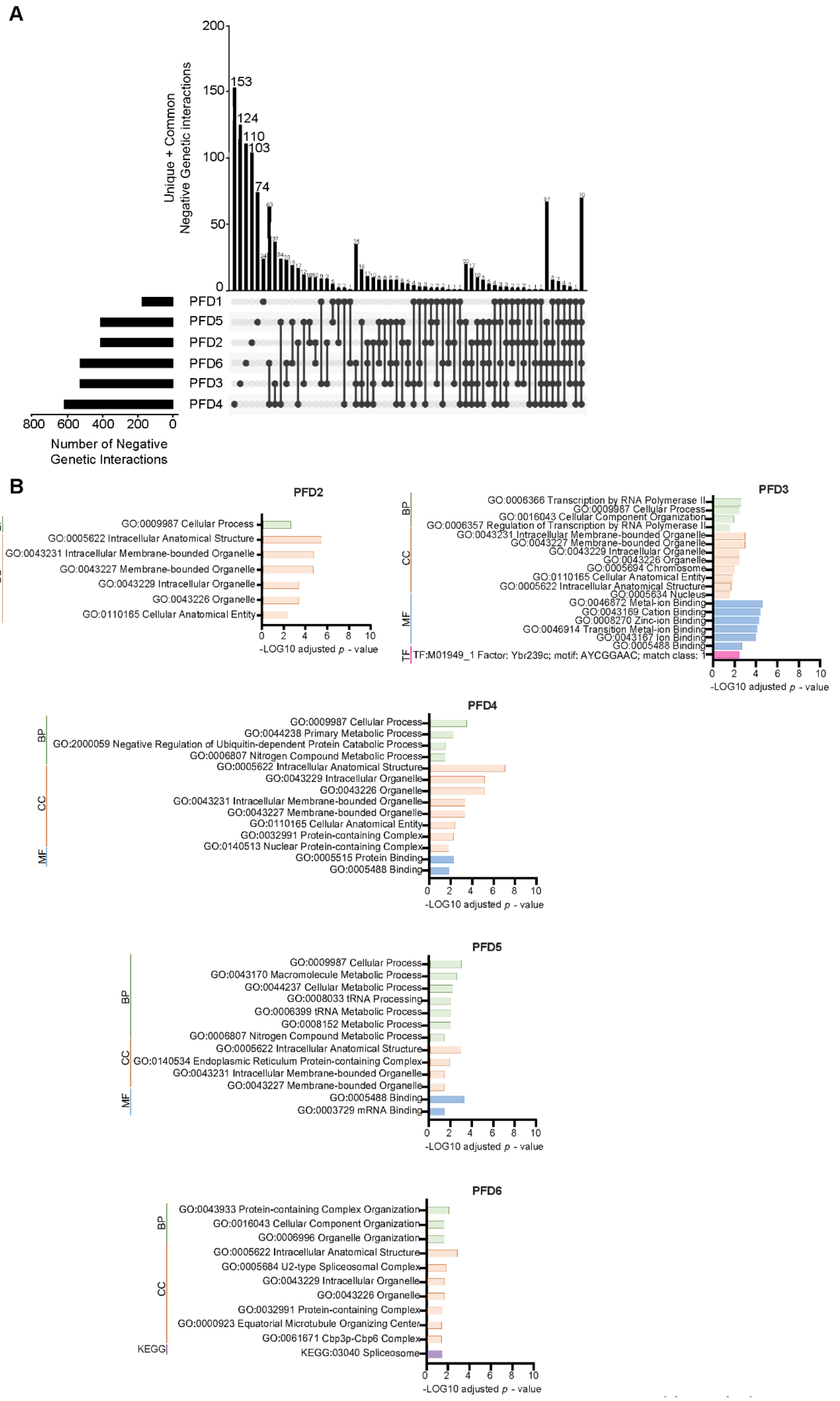

Fig. S4.; Related to Fig. 3. Deletions of prefoldin subunits have distinct negative genetic interaction partners. **A** UpSet plot illustrating the number of genes that when deleted result in a negative genetic interaction only with one prefoldin subunit (unique negative genetic interaction). **B** Gene ontology analysis of overrepresented terms within unique negative genetic interaction partners. No enrichment of GO terms was identified for negative genetic interactions with *Δpfd1*.
