# Supplementary material for: Prefoldin 2 contributes to mitochondrial morphology and function
Source: BMC Biol. 2023 Sep 12;21:193. doi: 10.1186/s12915-023-01695-y (PMC10496292; doi:10.1186/s12915-023-01695-y)
Supplement: Supplementary file 5 — Additional file 5: (Fig. S5.; Related to Fig. 3). Overexpression of mitochondrial genes in Δpfd2. A String network of mitochondrial genes that when deleted result in negative genetic interaction with Δpfd2. Marked in red are genes that grouped into the GO term “Mitochondrial membrane” (GO: 0031966). B Wildtype cells were transformed with an empty vector. Δpfd2 cells were transformed with an empty vector or a plasmid that harbored mitochondrial genes that were identified as negative genetic interactors with Δpfd2. The genes expression was under control of the constitutive TEF1 promoter. Transformed strains were grown on selective minimal medium that contained glycerol at 25°C to the logarithmic growth phase and shifted to 42°C for 6 h. Cell viability was assessed by propidium iodide staining and analyzed by flow cytometry. The data are presented as the mean ± SEM. n = 4. *p < 0.05. C Wildtype cells and cells that lacked PFD2 were transformed with Mas1-HA. Transformed cells were grown on selective minimal medium that contained glycerol at 25°C to the logarithmic growth phase and shifted to 42°C for 6 h. Insoluble proteins were obtained from the whole cell lysate by ultracentrifugation. Fractions (T, total cell extract 42°C; P, pellet; S, supernatant; T’, total cell extract 25°C) were separated on SDS-PAGE and analyzed by Western blot using specific antibodies. Uncropped blots are presented as source data in the Additional file 13. D Mas1-HA protein levels were quantified, and levels were normalized to Rpl17 protein levels. the representative blot shows two different yeast transformants (Col1 and Col2) for wildtype and Δpfd2 strains. The data are expressed as the mean ± SEM. n = 4 (for WT + MAS1-HA),n = 3 (for Δpfd2 + MAS1-HA). WT, wild type. [file 12915_2023_1695_MOESM5_ESM.pdf]

Additional file 5

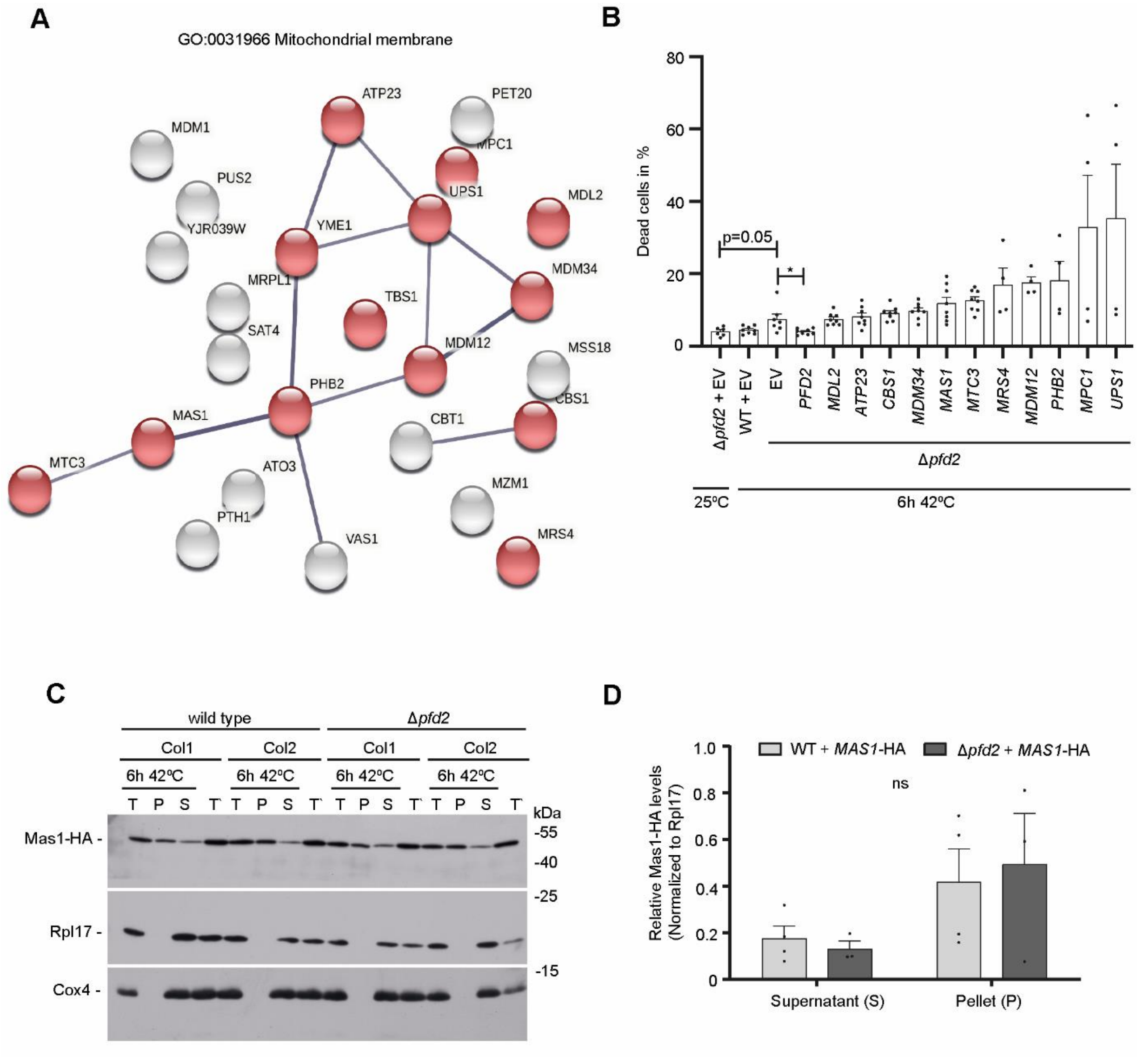

Fig. S5.; Related to Fig. 3. Overexpression of mitochondrial genes in Δ*pfd2*. **A** String network of mitochondrial genes that when deleted result in negative genetic interaction with *Δpfd2*. Marked in red are genes that grouped into the GO term "Mitochondrial membrane" (GO: 0031966). **B** Wildtype cells were transformed with an empty vector. Δ*pfd2* cells were transformed with an empty vector or a plasmid that harbored mitochondrial genes that were identified as negative genetic interactors with Δ*pfd2*. The genes expression was under control of the constitutive *TEF1* promoter. Transformed strains were grown on selective minimal

medium that contained glycerol at 25°C to the logarithmic growth phase and shifted to 42°C for 6 h. Cell viability was assessed by propidium iodide staining and analyzed by flow cytometry. The data are presented as the mean ± SEM. $n = 4$. $*p < 0.05$. **C** Wildtype cells and cells that lacked *PFD2* were transformed with Mas1-HA. Transformed cells were grown on selective minimal medium that contained glycerol at 25°C to the logarithmic growth phase and shifted to 42°C for 6 h. Insoluble proteins were obtained from the whole cell lysate by ultracentrifugation. Fractions (T, total cell extract 42°C; P, pellet; S, supernatant; T', total cell extract 25°C) were separated on SDS-PAGE and analyzed by Western blot using specific antibodies. Uncropped blots are presented as source data in the Additional file 13. **D** Mas1-HA protein levels were quantified, and levels were normalized to Rpl17 protein levels. the representative blot shows two different yeast transformants (Col1 and Col2) for wildtype and Δ*pfd2* strains. The data are expressed as the mean ± SEM. $n = 4$ (for WT + *MAS1*-HA), $n = 3$ (for Δ*pfd2* + *MAS1*-HA). WT, wild type.
