# Supplementary material for: Prefoldin 2 contributes to mitochondrial morphology and function
Source: BMC Biol. 2023 Sep 12;21:193. doi: 10.1186/s12915-023-01695-y (PMC10496292; doi:10.1186/s12915-023-01695-y)
Supplement: Supplementary file 6 — Additional file 6: (Fig. S6; Related to Fig. 4). Heat shock of Δpfd2 cells does not result in significant changes on the proteome level compared to wildtype cells. A Cells were grown in complete minimal medium to logarithmic growth phase at 25°C. The mRNA levels of the indicated genes were analyzed by quantitative real time PCR. The data are expressed as relative levels compared with the geometric mean of the house keeping genes ACT1, TDH1, and ALG9. n = 3. ***p < 0.001, **p< 0.01, *p < 0.05. ns, not significant. B-F Comparison of changes in proteome of Δpfd2 compared with wildtype cells at 37°C growth temperature. Volcano plot showing all identified proteins that localize to mitochondria (light pink circles in B), mitochondrial ribosome (dark pink circle in C), cytosolic ribosome (green circle in D), chaperones and protein folding (yellow circle in E), and proteasome (brown circle in F). WT, wild type. [file 12915_2023_1695_MOESM6_ESM.pdf]

Additional file 6

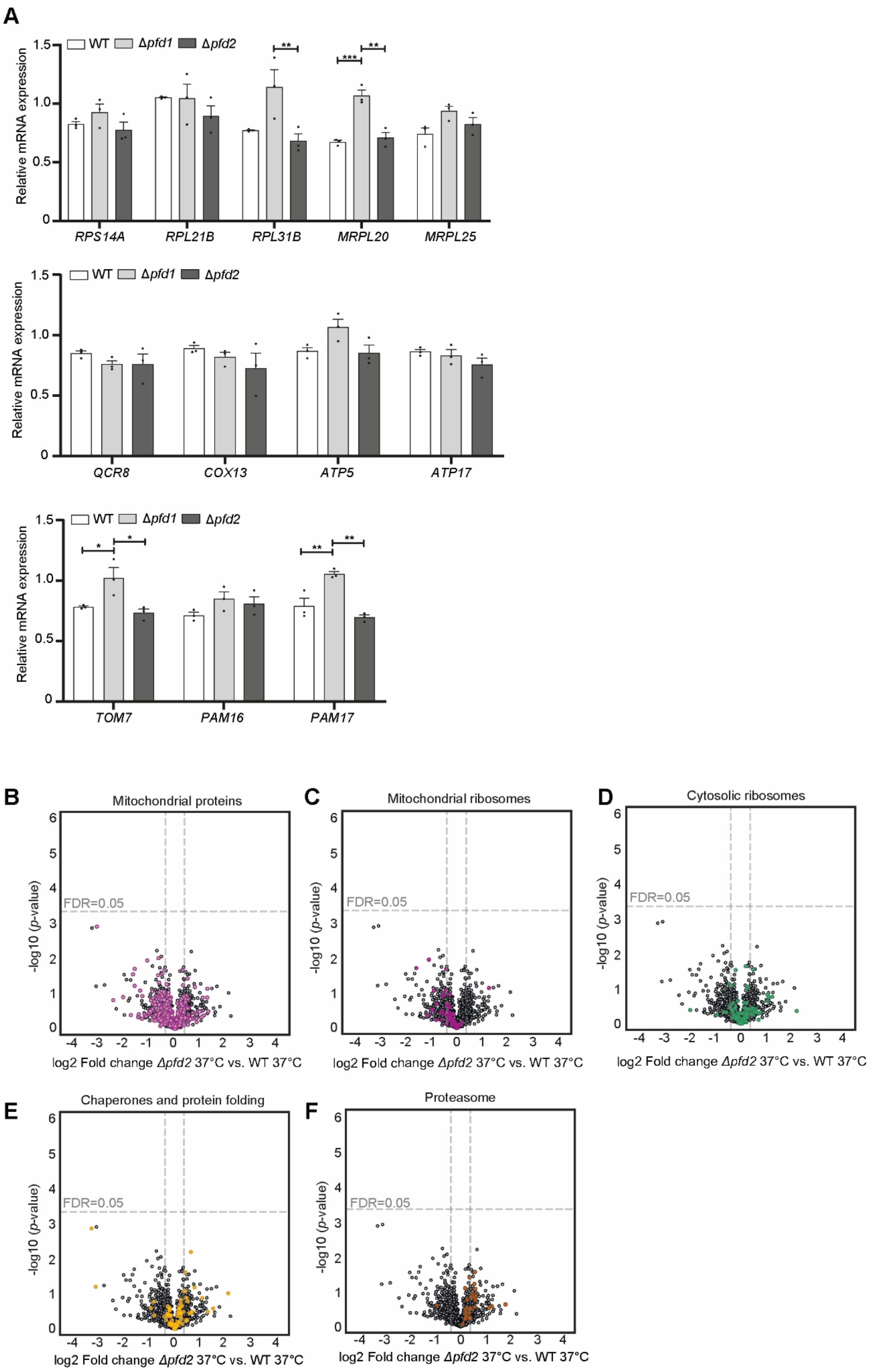

Fig. S6; Related to Fig. 4. Heat shock of Δ*pfd2* cells does not result in significant changes on the proteome level compared to wildtype cells. **A** Cells were grown in complete minimal medium to logarithmic growth phase at 25°C. The mRNA levels of the indicated genes were analysed by quantitative real time PCR. The data are expressed as relative levels compared with the geometric mean of the house keeping genes *ACT1*, *TDH1*, and *ALG9*. $n = 3$. ***$p < 0.001$, **$p < 0.01$, *$p < 0.05$. ns, not significant. **B-F** Comparison of changes in proteome of Δ*pfd2* compared with wildtype cells at 37°C growth temperature. Volcano plot showing all identified proteins that localize to mitochondria (light pink circles in B), mitochondrial ribosome (dark pink circle in C), cytosolic ribosome (green circle in D), chaperones and protein folding (yellow circle in E), and proteasome (brown circle in F). WT, wild type.
