# Supplementary material for: Prefoldin 2 contributes to mitochondrial morphology and function
Source: BMC Biol. 2023 Sep 12;21:193. doi: 10.1186/s12915-023-01695-y (PMC10496292; doi:10.1186/s12915-023-01695-y)
Supplement: Supplementary file 7 — Additional file 7: (Fig. S7; Related to Fig. 4). Comparison of heat shock response in wildtype cells and Δpfd2 cells. A, B Proteomes of wildtype (A) or Δpfd2 (B) cells grown at 25°C and shifted to 37°C for 4h were compared. Volcano plots showing proteins with significantly increased (blue circles) and decreased (red circles) protein abundance in wildtype cells 37°C vs. 25°C or Δpfd2 cells 37°C vs. 25°C. Adj. P-value of <0.05 (FDR = 0.05) and fold change of 1.3 was considered as statistical significant change. Non-significant proteins are shown in gray circles. C-L Volcano plots showing all identified proteins that localize to mitochondria (light pink circles in C, H), mitochondrial ribosome (dark pink circle in D, I), cytosolic ribosome (green circle in E, J), chaperones and protein folding (yellow circle in F, K), and proteasome (brown circle in G, L). C-G Comparison of changes in proteome of wildtype cells grown at 37°C with wildtype cells grown at 25°C. H-L Comparison of changes in proteome of Δpfd2 cells grown at 37°C with Δpfd2 cells grown at 25°C. WT, wild type. [file 12915_2023_1695_MOESM7_ESM.pdf]

Additional file 7

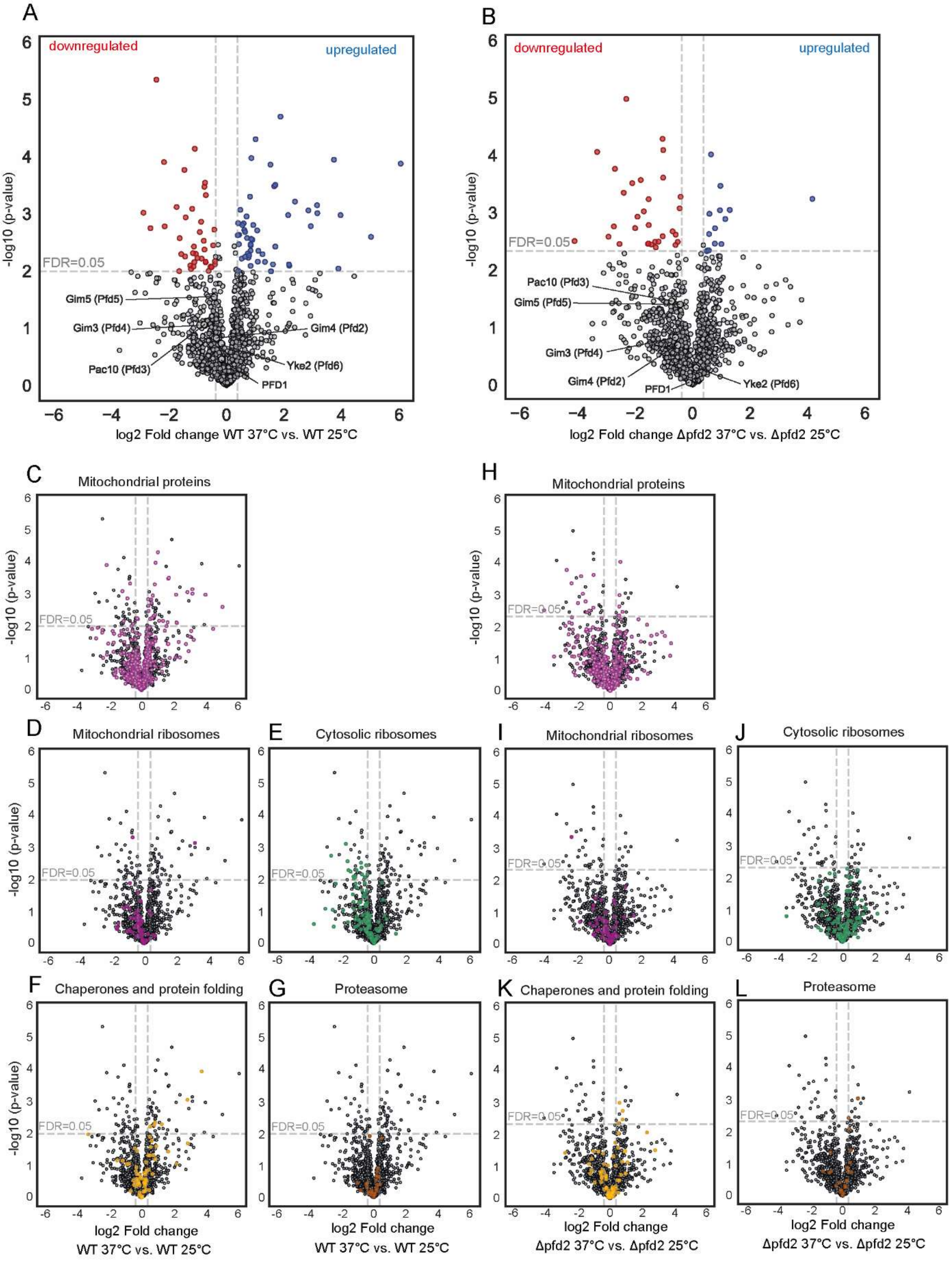

Fig. S7; Related to Fig. 4. Comparison of heat shock response in wildtype cells and Δ*pfd2* cells. **A, B** Proteomes of wildtype (A) or Δ*pfd2* (B) cells grown at 25°C and shifted to 37°C for 4h were compared. Volcano plots showing proteins with significantly increased (blue circles) and decreased (red circles) protein abundance in wildtype cells 37°C vs. 25°C or Δ*pfd2* cells 37°C vs. 25°C. Adj. *P*-value of <0.05 (FDR = 0.05) and fold change of 1.3 was considered as statistical significant change. Non-significant proteins are shown in gray circles. **C-L** Volcano plots showing all identified proteins that localize to mitochondria (light pink circles in C, H), mitochondrial ribosome (dark pink circle in D, I), cytosolic ribosome (green circle in E, J), chaperones and protein folding (yellow circle in F, K), and proteasome (brown circle in G, L). **C-G** Comparison of changes in proteome of wildtype cells grown at 37°C with wildtype cells grown at 25°C. **H-L** Comparison of changes in proteome of Δ*pfd2* cells grown at 37°C with Δ*pfd2* cells grown at 25°C. WT, wild type.
