# Supplementary material for: Prefoldin 2 contributes to mitochondrial morphology and function
Source: BMC Biol. 2023 Sep 12;21:193. doi: 10.1186/s12915-023-01695-y (PMC10496292; doi:10.1186/s12915-023-01695-y)
Supplement: Supplementary file 8 — Additional file 8: (Fig. S8; Related to Fig. 4). Cellular responses of wildtype cells upon heat shock. A Gene ontology enrichment of proteins with significantly downregulated (upper panel) or upregulated (lower panel) protein abundance in wildtype cells grown at 37°C compared with wildtype grown at 25°C. Values next to the right side of the bars indicate the numbers of proteins with the certain GO term. B Interaction network of all proteins significantly downregulated. In pink are proteins localized to mitochondria and in green are proteins of the cytosolic ribosome marked. Other proteins are shown in gray. C Interaction network of all proteins significantly upregulated. In pink are proteins with mitochondrial localization, in yellow are proteins identified as chaperones. Other proteins are shown in gray. WT, wild type. [file 12915_2023_1695_MOESM8_ESM.pdf]

Additional file 8

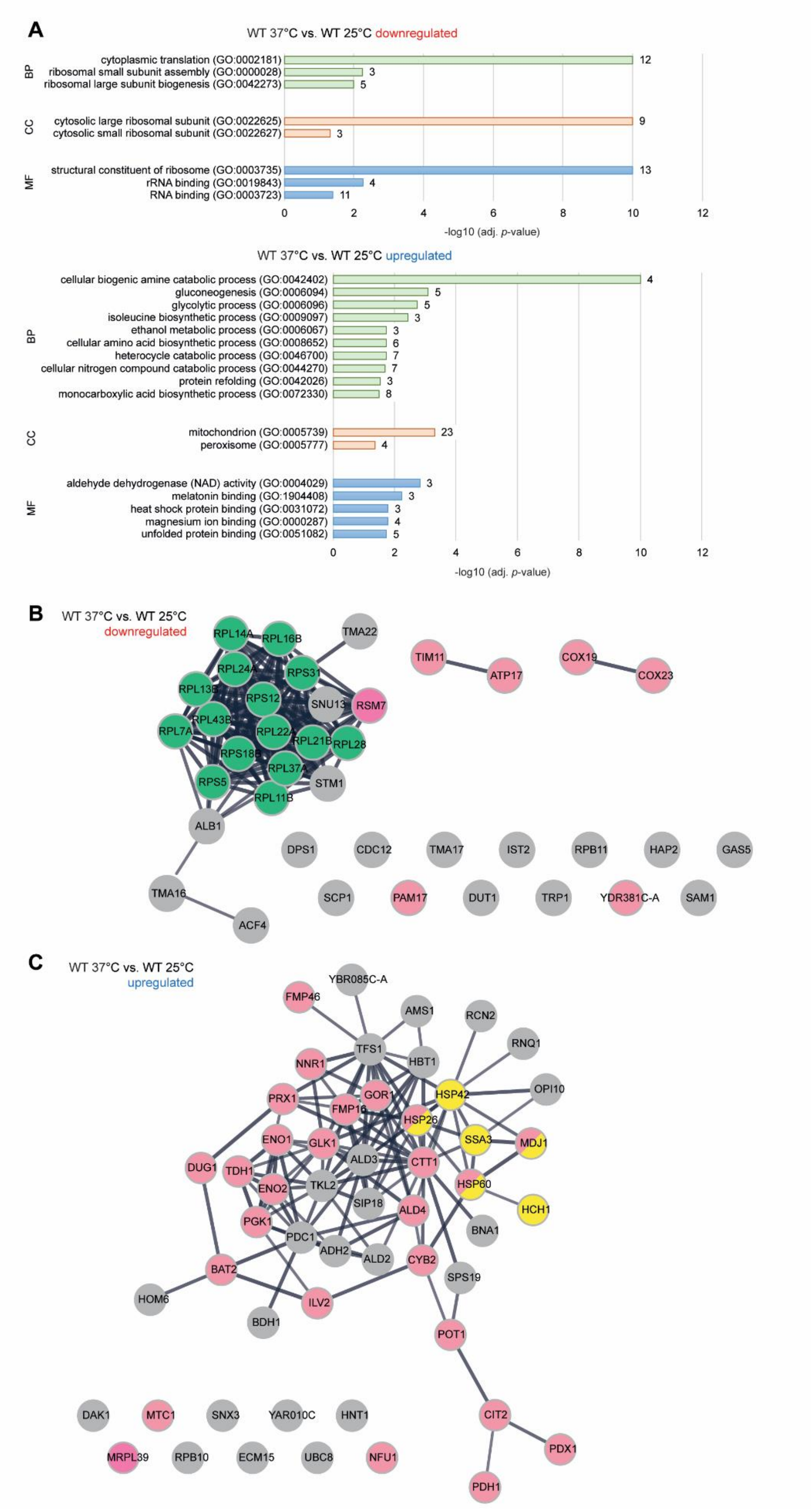

Fig. S8; Related to Fig. 4. Cellular responses of wildtype cells upon heat shock. **A** Gene ontology enrichment of proteins with significantly downregulated (upper panel) or upregulated (lower panel) protein abundance in wildtype cells grown at 37°C compared with wildtype grown at 25°C. Values next to the right side of the bars indicate the numbers of proteins with the certain GO term. **B** Interaction network of all proteins significantly downregulated. In pink are proteins localized to mitochondria and in green are proteins of the cytosolic ribosome marked. Other proteins are shown in gray. **C** Interaction network of all proteins significantly upregulated. In pink are proteins with mitochondrial localization, in yellow are proteins identified as chaperones. Other proteins are shown in gray. WT, wild type.
