# Supplementary material for: Prefoldin 2 contributes to mitochondrial morphology and function
Source: BMC Biol. 2023 Sep 12;21:193. doi: 10.1186/s12915-023-01695-y (PMC10496292; doi:10.1186/s12915-023-01695-y)
Supplement: Supplementary file 9 — Additional file 9: (Fig. S9; Related to Fig. 4). Cellular responses of Δpfd2 cells upon heat shock. A Gene ontology enrichment of proteins with significantly downregulated (upper panel) or upregulated (lower panel) protein abundance in Δpfd2 cells grown at 37°C compared with Δpfd2 grown at 25°C. Values next to the right side of the bars indicate the numbers of proteins with the certain GO term. B Interaction network of all proteins significantly downregulated. In pink are proteins localized to mitochondria. Other proteins are shown in gray. C Interaction network of all proteins significantly upregulated. In pink are proteins with mitochondrial localization, in yellow are proteins identified as chaperones, and in brown a protein of the proteasome is shown. Other proteins are shown in gray. [file 12915_2023_1695_MOESM9_ESM.pdf]

Additional file 9

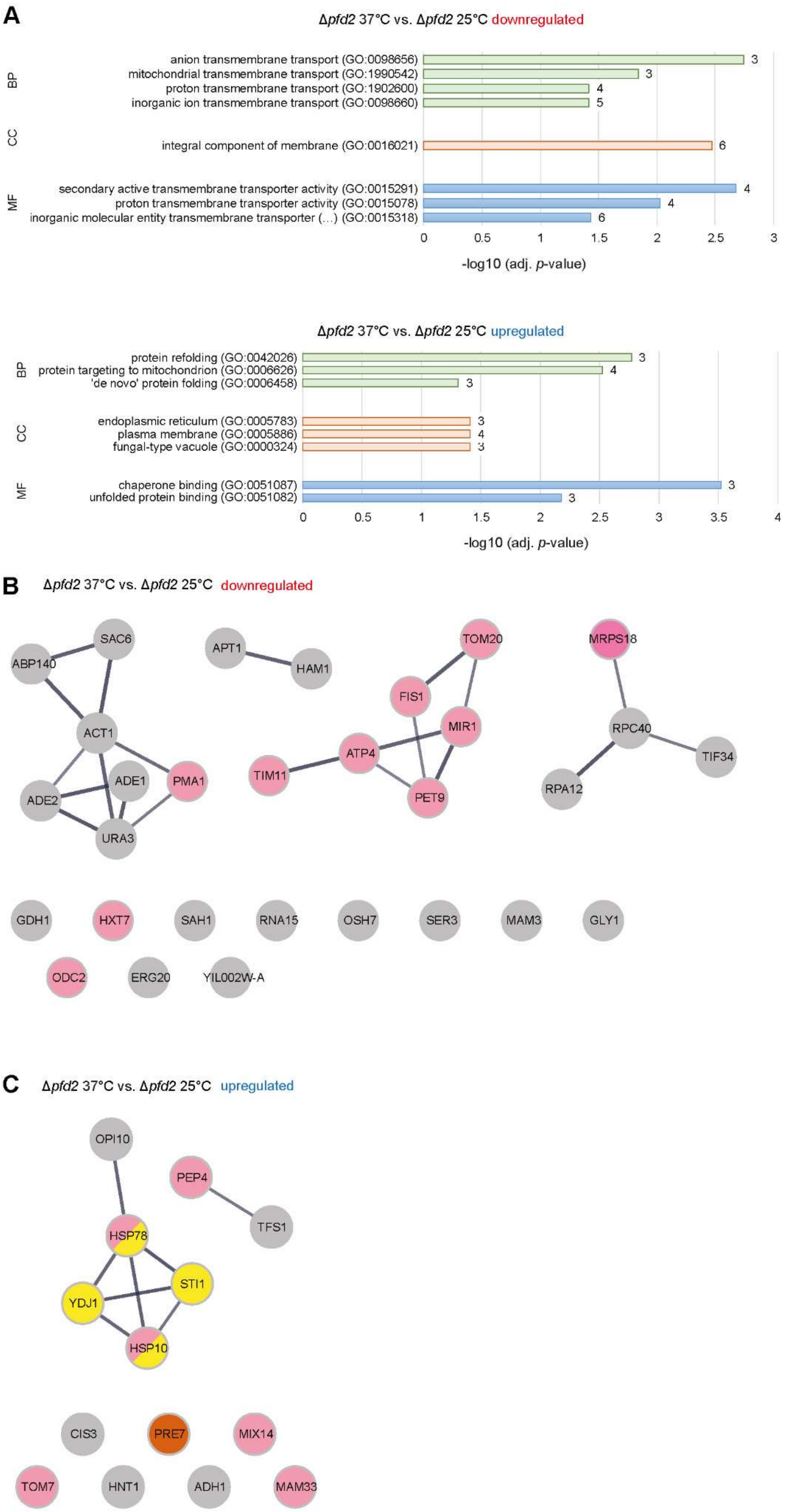

Fig. S9; Related to Fig. 4. Cellular responses of Δ*pfd2* cells upon heat shock. **A** Gene ontology enrichment of proteins with significantly downregulated (upper panel) or upregulated (lower panel) protein abundance in Δ*pfd2* cells grown at 37°C compared with Δ*pfd2* grown at 25°C. Values next to the right side of the bars indicate the numbers of proteins with the certain GO term. **B** Interaction network of all proteins significantly downregulated. In pink are proteins localized to mitochondria. Other proteins are shown in gray. **C** Interaction network of all proteins significantly upregulated. In pink are proteins with mitochondrial localization, in yellow are proteins identified as chaperones, and in brown a protein of the proteasome is shown. Other proteins are shown in gray.
