# Supplementary material for: Prefoldin 2 contributes to mitochondrial morphology and function
Source: BMC Biol. 2023 Sep 12;21:193. doi: 10.1186/s12915-023-01695-y (PMC10496292; doi:10.1186/s12915-023-01695-y)
Supplement: Supplementary file 13 — Additional file 13. Source data presenting uncropped western blots. [file 12915_2023_1695_MOESM13_ESM.pdf]

Additional File 13

Figure 6A

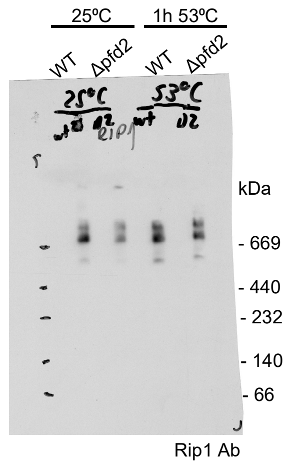

Figure 6B

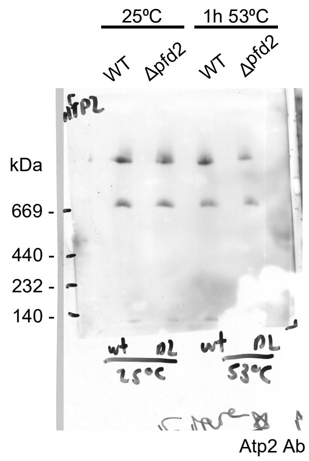

Figure 6C

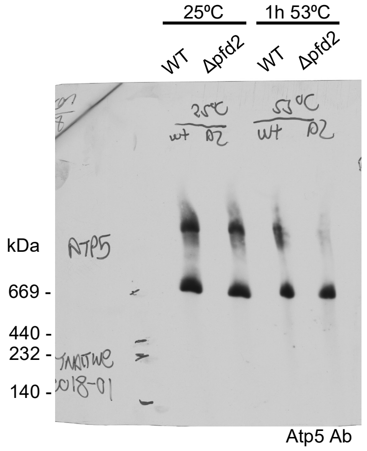

Figure 6D

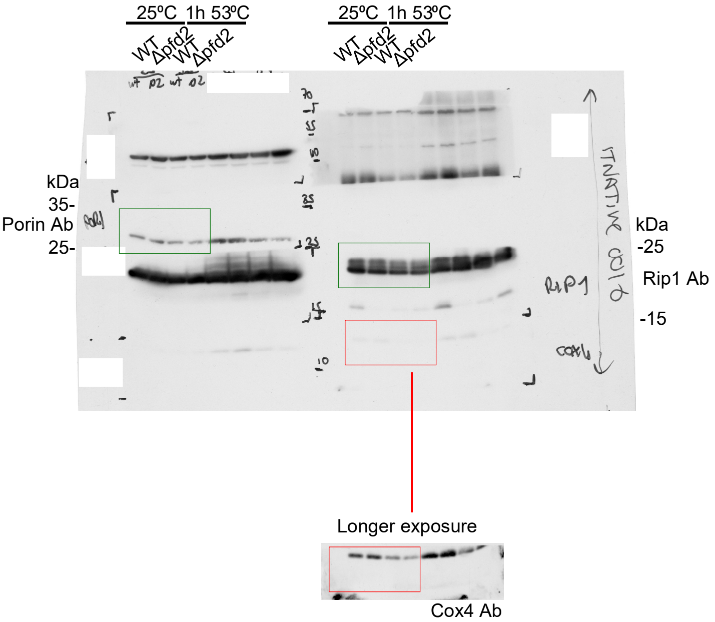

Figure 6E

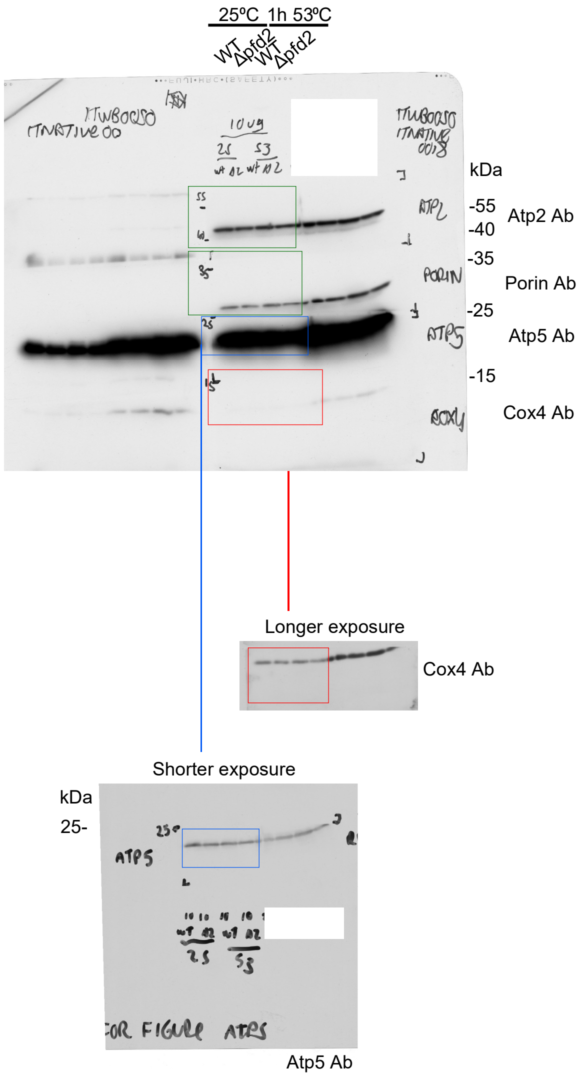

After protein transfer, membranes were cut and their fragments were incubated with antibodies against indicated proteins

Additional File 13

Figure 6G

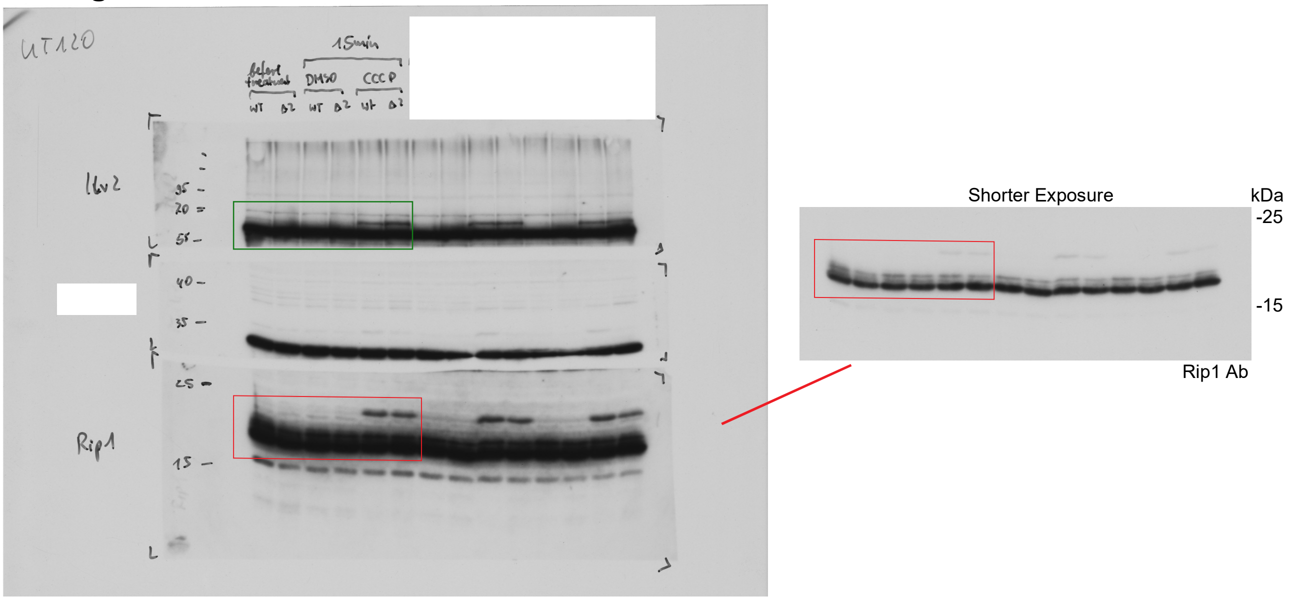

Figure 6H

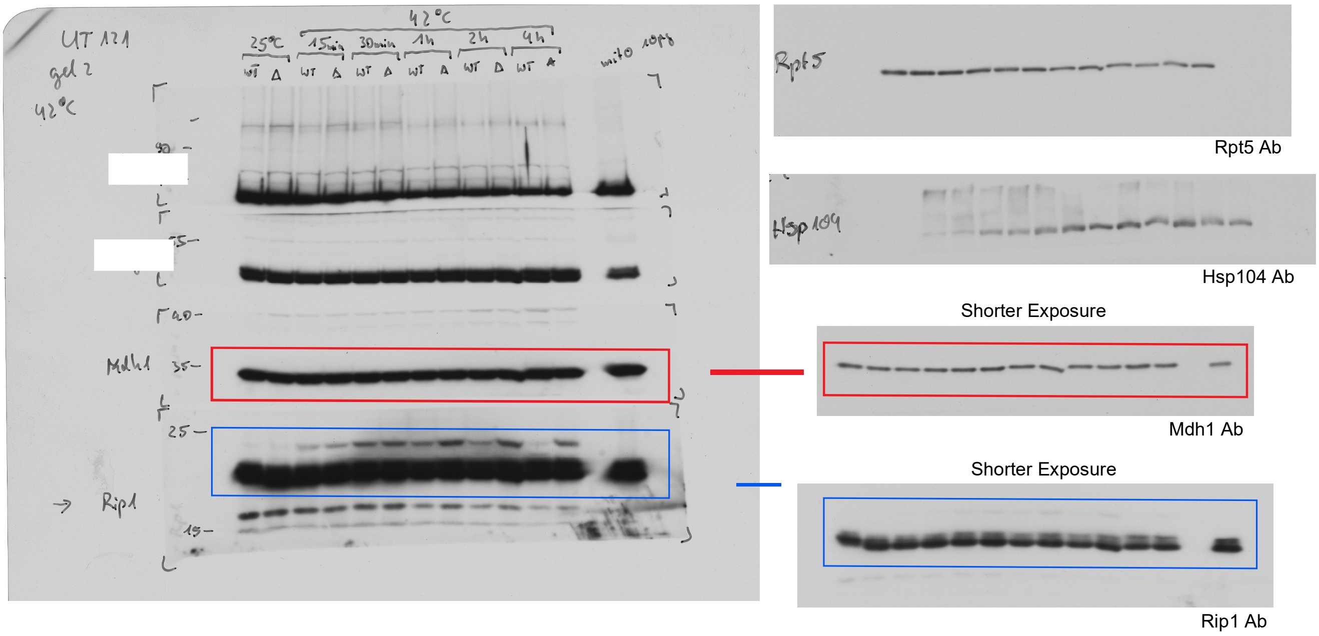

Figure 6I

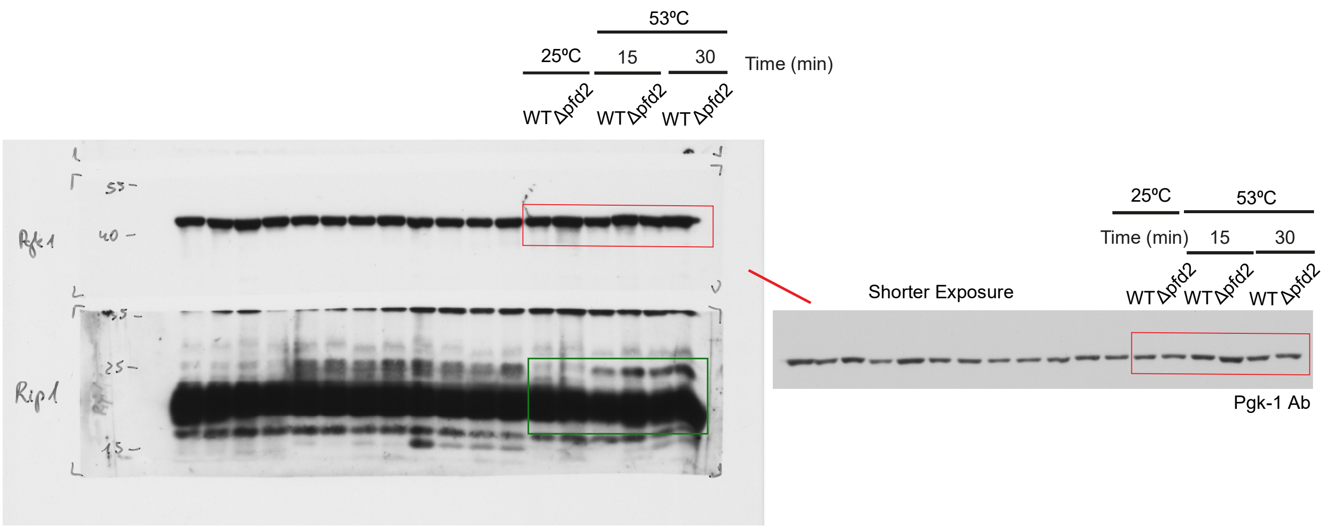

After protein transfer, membranes were cut and their fragments were incubated with antibodies against indicated proteins

Additional File 13

Figure 7A

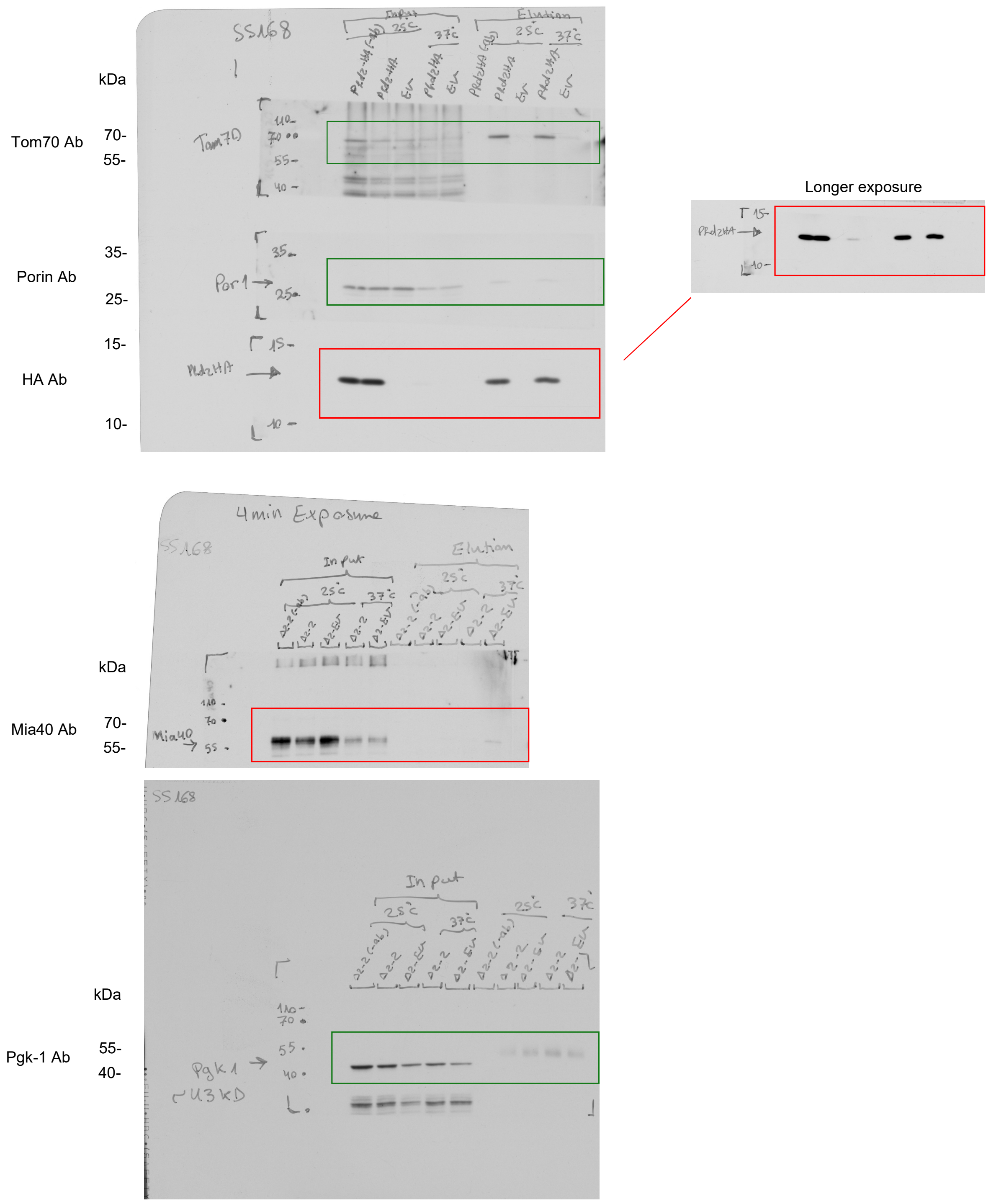

After protein transfer, membranes were cut and their fragments were incubated with antibodies against indicated proteins

Additional File 13

Additional File 2 FigureS2C

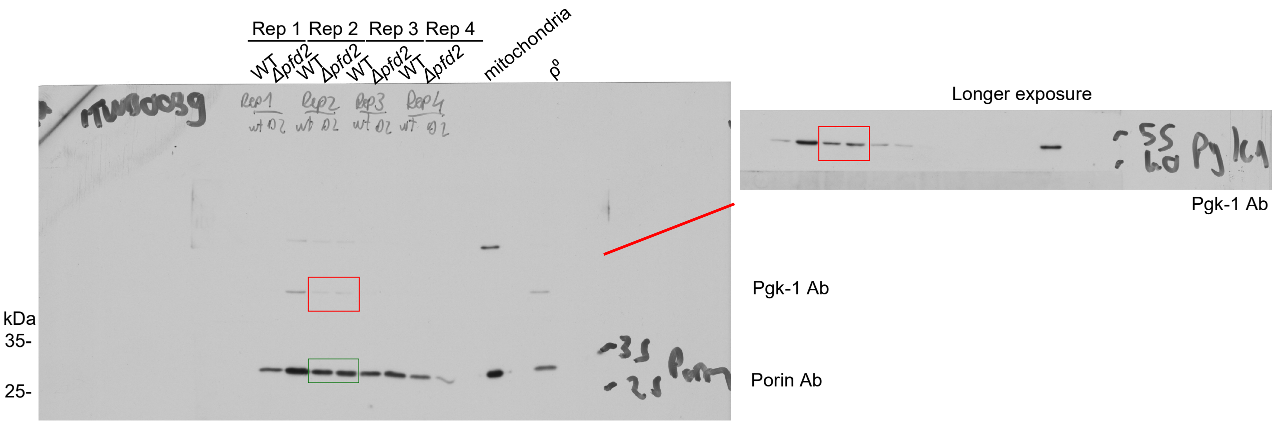

Additional File 2 FigureS2E

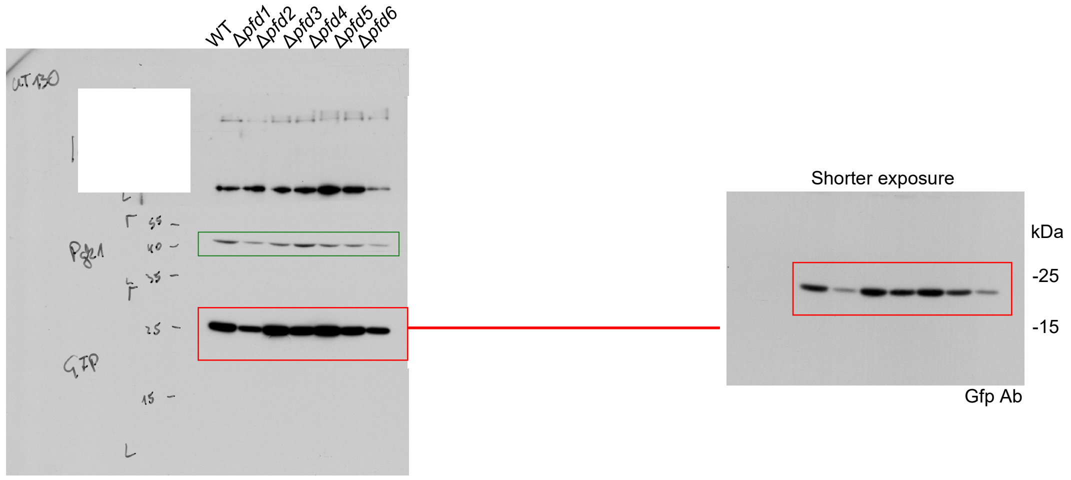

Additional File 2 Figure S2G

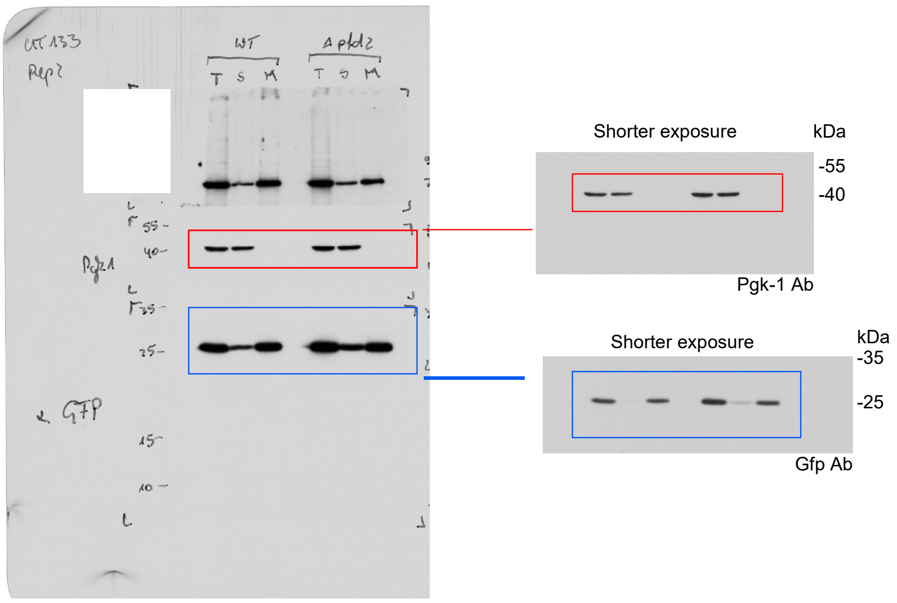

After protein transfer,membranes were cut and their fragments were incubated with antibodies against indicated proteins

Additional File 13

Additional File 5 Figure S5C

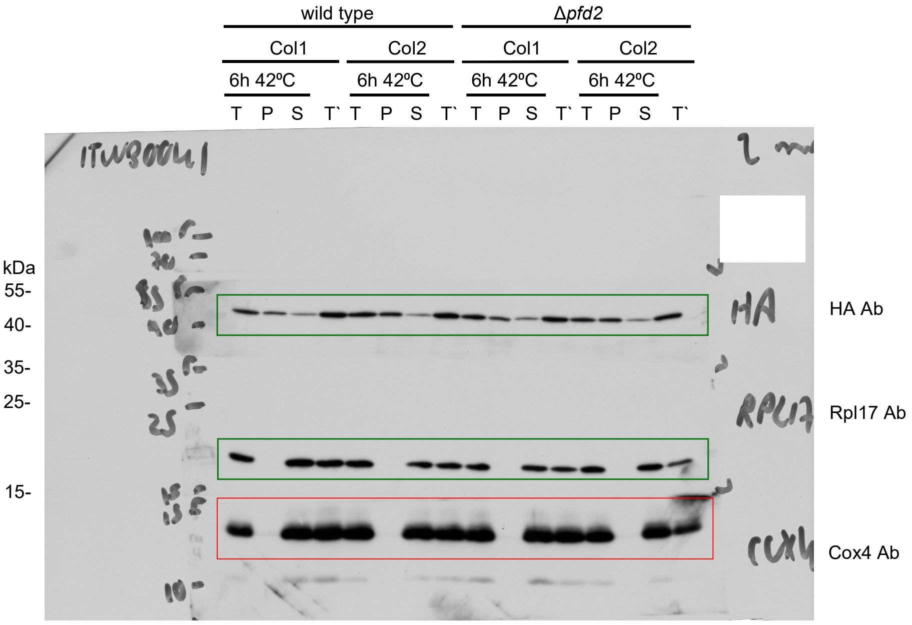

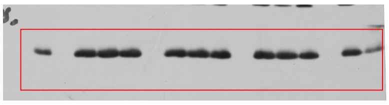
Shorter exposure

After protein transfer,membranes were cut and
their fragments were incubated with antibodies against indicated proteins
